# Nanoparticle-Guided Brain Drug Delivery: Expanding the Therapeutic Approach to Neurodegenerative Diseases

**DOI:** 10.3390/pharmaceutics13111897

**Published:** 2021-11-08

**Authors:** Claudia Riccardi, Filomena Napolitano, Daniela Montesarchio, Simone Sampaolo, Mariarosa Anna Beatrice Melone

**Affiliations:** 1 Department of Chemical Sciences, University of Naples Federico II, Via Cintia 21, I-80126 Naples, Italy; claudia.riccardi@unina.it (C.R.); daniela.montesarchio@unina.it (D.M.); 2 Department of Advanced Medical and Surgical Sciences, 2nd Division of Neurology, Center for Rare Diseases and InterUniversity Center for Research in Neurosciences, University of Campania Luigi Vanvitelli, Via Sergio Pansini, 5, I-80131 Naples, Italy; filomena.napolitano@unicampania.it (F.N.); simone.sampaolo@unicampania.it (S.S.); 3 Sbarro Institute for Cancer Research and Molecular Medicine, Center for Biotechnology, Temple University, Philadelphia, PA 19122-6078, USA

**Keywords:** neurodegenerative diseases, blood–brain barrier, brain targeting, nanoformulations, drug delivery systems, targeted delivery, nose-to-brain delivery

## Abstract

Neurodegenerative diseases (NDs) represent a heterogeneous group of aging-related disorders featured by progressive impairment of motor and/or cognitive functions, often accompanied by psychiatric disorders. NDs are denoted as ‘protein misfolding’ diseases or proteinopathies, and are classified according to their known genetic mechanisms and/or the main protein involved in disease onset and progression. Alzheimer’s disease (AD), Parkinson’s disease (PD) and Huntington’s disease (HD) are included under this nosographic umbrella, sharing histopathologically salient features, including deposition of insoluble proteins, activation of glial cells, loss of neuronal cells and synaptic connectivity. To date, there are no effective cures or disease-modifying therapies for these NDs. Several compounds have not shown efficacy in clinical trials, since they generally fail to cross the blood-brain barrier (BBB), a tightly packed layer of endothelial cells that greatly limits the brain internalization of endogenous substances. By engineering materials of a size usually within 1–100 nm, nanotechnology offers an alternative approach for promising and innovative therapeutic solutions in NDs. Nanoparticles can cross the BBB and release active molecules at target sites in the brain, minimizing side effects. This review focuses on the state-of-the-art of nanoengineered delivery systems for brain targeting in the treatment of AD, PD and HD.

## 1. Introduction: Neurodegenerative Diseases

In recent years, great advances have been made in understanding the genetic, molecular, and biochemical mechanisms of neurodegenerative diseases (NDs), a group of disorders of the central nervous system (CNS) featured by extra- and intra-cellular accumulation of misfolded proteins, as well as progressive dysfunction, degradation or death of neurons. However, surviving neurons show remarkable morphological changes in the size and shape of the nucleus and chromatin condensation [1].

Protein misfolding is the main cause of a series of strictly connected events, first and foremost, the failure of the ubiquitin-proteasome system (UPS), followed by the collapse of autophagy. Both events lead to oxidative stress, mitochondrial energy deficiency, dysfunction of neurotrophins, and activation of neuroinflammation, as well as extensive formation of free radicals. Concomitantly, dysfunctions of the neuronal Golgi apparatus and axonal transport can frequently occur [1].

Alzheimer’s disease (AD), Parkinson’s disease (PD), and Huntington’s disease (HD) are considered worldwide as the main neurodegenerative disorders [2,3], sharing several similarities at the subcellular and molecular level such as synaptic abnormalities, deposition of misfolded proteins in the brain, activation of glial cells, and loss of neuronal connectivity of synaptic circuits. All these diseases usually appear in adulthood as slowly progressive disorders, mainly affecting motor, cognitive, and mental functions [4,5].

### 1.1. Alzheimer’s Disease

Alzheimer’s disease (AD) is the most prevalent cause of dementia, with the number of patients expected to reach more than 150 millions by mid-century worldwide. AD is a slowly progressive ND, which gradually impairs activities of daily living and social functioning in affected people [2,6,7,8].

The development of AD symptoms is very insidious: it takes many years before patients begin to show memory impairment exceeding that typically observed for their age group—a stage with an alarming prognosis known as mild cognitive impairment (MCI)—and many more years before their cognitive abilities decline to a functionally disabling degree, with loss of spatial and temporal orientation, as well as verbal fluency [9,10].

In most cases, AD is late-onset and occurs sporadically, but some early-onset familial forms have been described, mainly linked to three causative genes, which are *APP, PSNE1,* and *PSEN2*, which respectively code amyloid precursor protein (APP), presenilin-1 (PSNE1), and presenilin-2 (PSNE2) proteins. In addition, several genetic risk factors, including the ε4 allele of apolipoprotein E (ApoE), have been reported [11].

**Figure 1:**
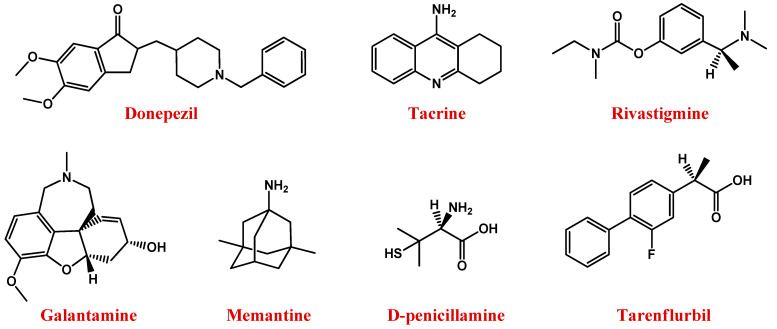
Molecular structures of main anti-AD drugs.

Currently, there is no definitive treatment for AD, and the available therapeutic interventions (Figure 1) are aimed at managing the disease, i.e., mitigating or halting the associated symptoms [12,13,14,15].

In sporadic AD, a reduction in brain weight, although not constant, is generally usual, as evidenced by macroscopic findings. More severe atrophy is evident in early-onset and familial AD. Diffuse gyral atrophy and ventricular dilatation—involving the temporal cortex, amygdala, hippocampus, and entorhinal cortex, without affecting the occipital lobe—are often found.

Microscopic neuropathological features of AD—detected on post-mortem brain examination—include intracellular neurofibrillary tangles (NFTs), and extracellular plaques of β-amyloid peptide (Aβ), both associated with neuronal loss and altered synaptic connectivity [16,17,18,19].

Under physiological conditions, microtubule-associated tau protein supports neuronal growth, but becomes cytotoxic when hyperphosphorylated, precipitating as paired helical filaments, i.e., NFTs. Tau aggregation affects neuronal axons and, consequently, causes neurodegeneration, with significant effects on AD pathogenesis and progression [20,21,22,23,24].

In turn, according to the Aβ cascade hypothesis, the formation of Aβ plaques is essentially generated from the catalytic cleavage of APP, a transmembrane glycoprotein composed of 770 amino acids, operated first by β-secretase and then by γ-secretase, respectively, at the N- and C-termini of the protein [25,26,27].

The deposition of the cleavage product of APP is ultimately the strong self-aggregating β-amyloid peptide of 42 amino acids, known as Aβ_1–42_ [28], which is neurotoxic both in vitro and in vivo [29]. Aβ monomers form dimers, then oligomers, protofibrils, and mature fibrils, finally resulting in the formation of Aβ aggregates [30].

Accumulation of Aβ_1–42_ promotes further pathological effects, such as disruption of synaptic connections with consequent neuronal damage and decreased release of cholinergic neurotransmitters. Indeed, the cholinergic system plays an important role in learning so that cholinergic dysfunctions are the main causes of memory impairments observed in AD patients [31,32]. Therefore, many treatment strategies for AD have been focused on the restoration of the cholinergic neurotransmission [33,34].

In this context, available drugs are mainly cholinesterase inhibitors (ChEIs)—including donepezil [35], tacrine [36], rivastigmine [37], and galantamine [38] (Figure 1) —which prevent acetylcholine breakdown, improving its bioavailability [33,34].

Memantine (Figure 1), an uncompetitive antagonist of glutamatergic *N*-methyl-d-aspartate (NMDA) receptors, represents a valuable therapeutic option for AD. Indeed, glutamate stimulates post-synaptic receptors involved in memory processes, while memantine decreases the excess of glutamate responsible for neuronal death in AD patients [39,40,41].

Other promising anti-AD agents are molecules preventing Aβ or tau aggregation, from small compounds [42,43,44] to peptides and monoclonal antibodies (mAbs) [14,44,45,46,47].

For example, based on the 17–21 residues of the Aβ peptide, i.e., LVFFA, Soto et al. designed a peptide inhibitor LPFFD, also known as iAβ5 [48,49]. Due to the substitution of valine with a proline residue, not able to fit in the β-sheet structure, iAβ5 exhibited a very low propensity to adopt a β-sheet conformation, preventing the interaction between Aβ molecules and the formation of β-sheet oligomers [48,50].

Moreover, residues 16–20 of the Aβ peptide, i.e., KLVFF, are crucial for the formation of β-sheet structures [51]. KLVFF peptide can bind its homologous sequence in Aβ, preventing its aggregation into amyloid fibrils [52,53,54].

An alternative strategy explored in AD treatment consists in the solubilization of preformed β-amyloid plaques, successfully realized with humanized mAbs or copper/zinc chelators. Indeed, oxidative damage – promoted by metals such as iron, zinc, copper, and aluminium – is one of the main causes of AD, since these metals interact with β-amyloids and promote their aggregation [55].

Therefore, the use of suitable chelators, such as the copper chelator d-penicillamine (Figure 1) [56,57], able to reduce the amount of metal in the brain, can represent a valid approach to AD treatment [58,59].

Further available anti-AD agents are non-steroidal anti-inflammatory drugs such as tarenflurbil (Figure 1), i.e., the pure R-enantiomer of flurbiprofen [60] and/or antioxidants [61], most of which are derived from natural sources.

For example, osmotin is a 24 kDa multifunctional plant-derived protein from tobacco (*Nicotiana tabacum*), with a remarkable neuroprotective effect, demonstrated in a mice model of AD [62,63,64].

However, all these available drug options are aimed at managing the disease, i.e., mitigating or halting AD associated symptoms, since no definitive cure exists [12,13,14,15].

### 1.2. Parkinson’s Disease

The second most common age-related neurodegenerative disorder and most common movement disorder is Parkinson’s disease (PD), with a prevalence of over 2% after the age of 65.

Pathological features of this ND are a reduced number of dopaminergic neurons in the substantia nigra pars compacta and the presence of Lewy bodies, i.e., eosinophilic intracellular inclusions formed by aggregates of the α-synuclein (SNCA) protein. These aggregates begin to form in the medulla and olfactory bulbs, spreading progressively—according to Braak’s six-stage description—to involve pons, midbrain, limbic lobe, amygdala, and neocortex [65,66,67,68]. The spread of Lewy bodies eventually leads to the dysfunction of other neurotransmitter systems, such as adrenergic, cholinergic, and serotonergic [69,70].

Owing to the geographical variability observed in PD incidence, this disease has been regarded for years as a purely sporadic disorder, mainly of environmental origin. The era of the recognized genetic contribution to the pathogenesis of PD began in the late 1990s, thanks to the Contursi family (from the namesake village in Campania, Italy), in which molecular genetic studies revealed in the gene locus 4q21-22 (termed *PARK1)* the first *SNCA*-associated PD [71]. Since this gene has been identified, more than twenty genes and several independent risk-associated variants have been linked to PD onset [72].

Several studies also demonstrated that the dysfunction of the UPS system plays a direct role in the pathogenesis of PD. Under physiological conditions, UPS is responsible for most of the protein turnover within cells. In PD, misfolded and aggregated SNCA impairs UPS function contributing to neuronal death [73].

It is now well accepted that PD is a complex multisystem ND, with both motor and non-motor signs and symptoms [74].

Since dopamine is involved in motor functions, its reduction results in brady-hypokinesia, rigidity, tremors, decreased balance, and gait difficulties, which are the cardinal features of the PD motor phenotype [75,76,77].

Non-motor signs—which are very prominent, especially in advanced PD stages—result from multiple neurotransmitter deficits in both the central and peripheral nervous systems, and significantly affect the quality of life of PD patients. These signs include: cognitive (dysexecutive syndrome, mild cognitive impairment to dementia), behavioural (hallucinations, delusions), mood depression, pain, dysautonomia (constipation, urgency, orthostatic hypotension), sleep, and vigilance disturbances [78,79]. Non-motor signs may also occur in the first stages of the disease, or precede the motor phase by several years, such as olfactory deficit due to olfactory nerve damage [80], or disturbances during rapid eye movement (REM) sleep, such as vivid dreams and/or nightmares, that may be responsible for self- or hetero-aggressive acts, or constipation.

Currently, the disease remains pathogenetically incurable, and only symptomatic approaches are available for PD patients, while no disease-modifying therapies have been described [81,82].

**Figure 2:**
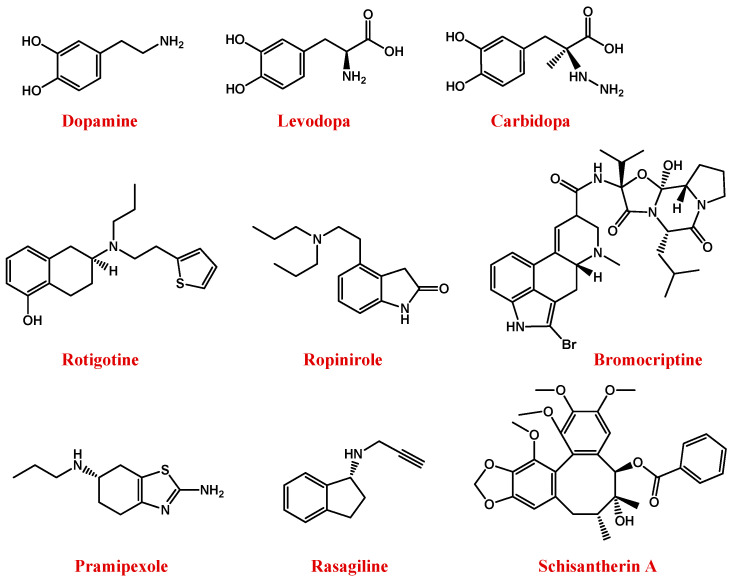
Molecular structures of main anti-PD drugs.

To restore the dopaminergic transmission, current approaches are essentially based on exogenous dopamine supply as such or administered as its levodopa (l-DOPA) precursor (Figure 2) [83,84].

Indeed, dopamine is not able to overcome the blood–brain barrier (BBB), due to its low lipid solubility and lack of specific transporters. In contrast, its natural l-DOPA precursor can cross the BBB to a certain extent and is then converted to dopamine in the brain, due to the DOPA decarboxylase enzyme [85,86]. However, when orally administered, l-DOPA is rapidly decarboxylated to dopamine; thus, the amount of drug effectively able to reach as such the CNS is very small. So, to maintain its effectiveness, the l-DOPA dosage should be enhanced, but this increased dose is often associated with severe side effects, such as depression, anxiety, insomnia, agitation, nausea, and vomiting [70,81,87]. Moreover, its long-term use is accompanied by adverse effects, such as tardy action and disabling dyskinesia termed “Levodopa-induced dyskinesia” [88].

To maintain the l-DOPA efficiency avoiding high doses or high dosage frequency, another effective possibility consists in its co-administration with carbidopa (Figure 2), an inhibitor of the DOPA decarboxylase enzyme, able to prevent l-DOPA metabolism at the periphery [81,82].

However, disadvantages associated with the clinical use of l-DOPA stimulated the search for novel anti-PD drugs. In this context, rotigotine—a non-ergot-derived D3/D2/D1 agonist (Figure 2)—proved to have neuroprotective properties and lighten the motor symptoms of PD [89,90,91]. Alternative dopamine agonists are ropinirole, bromocriptine, and pramipexole (Figure 2) [91].

Ropinirole is a non-ergoline D2/D3 dopamine receptor agonist able to specifically bind D2-receptors in the striatum and substantia nigra [92,93].

Bromocriptine is a semi-synthetic ergopeptine derivative and a potent dopamine receptor agonist able to stimulate the striatal D2 non-adenyl cyclase-linked dopamine receptors [94].

Pramipexole is a non-ergot dopamine agonist especially used in association with l-DOPA or monoamine oxidase B (MAO-B) enzyme inhibitors [95,96].

Indeed, MAO-B is the main enzyme involved in the metabolic degradation of dopamine, reducing its available amount in the brain. Thus, the inhibition of its activity, using for example rasagiline (Figure 2), represents a useful approach to restore dopamine levels [97,98,99].

Urocortin, a corticotrophin-releasing hormone-related peptide, has recently been proposed as a cytoprotectant for cultured hippocampal neurons, cerebellar granule cells, and GABAergic neurons [100,101].

Iron accumulation in substantia nigra pars compacta has been proven to be a pathophysiological feature of PD, which could induce the death of dopaminergic neurons, reactive oxygen species (ROS) up-regulation, and further loss of motor control [102,103]. Thus, the term “iron-chelation therapy” generally refers to the reduction of abnormal iron accumulation in substantia nigra pars compacta [104].

As for AD, antioxidant agents could be effective also in PD treatment. Most antioxidant compounds are obtained from natural sources as schisantherin A (Figure 2), a major dibenzocyclooctadiene lignan isolated from the fruit of *Schisandra chinensis*, with potential anti-PD properties [105,106].

### 1.3. Huntington’s Disease

Huntington’s disease (HD) is a rare neurodegenerative disorder that primarily affects basal ganglia neurons (caudate nucleus and putamen) with striatal medium spiny neurons (composed of GABAergic neurons) almost completely lost in the advanced stages of this disease. Early dysfunction with subsequent loss of cortical neurons is also prominent and consistent with a decrease in brain weight [107,108,109,110,111].

HD is caused by dominant mutations in the exon 1 of *Huntingtin* (*HTT*) gene encoding the HTT protein. The mutation consists of an abnormal repetition of the CAG nucleotide triplet (40 or above), leading to the production of an altered protein with an abnormally long polyglutamine tract (polyQ) at the N-terminal extremity [112]. This polyQ segment induces the intracellular aggregation of the mutant HTT protein (mHTT) in the caudate nucleus and putamen of basal ganglia, ultimately causing cortico-striatal dysfunction and degeneration [113,114,115,116,117].

The typical signs and symptoms of HD—which generally become evident between the ages of 30 and 50, with some reported cases of juvenile forms of HD (onset before the age of 20) [118]—consist of a triad of motor, cognitive and psychiatric symptoms. The most typical sign of HD is represented by choreic movements—from ancient Greek: χορεία (‘dance’)—hence, this ND is also known as Huntington’s chorea.

Other motor symptoms include abnormal postures (dystonia), rigidity with slow voluntary movements (bradykinesia), and convulsions. Cognitive impairments are featured by a progressive slowing of ideational processes and deterioration of memory, that gradually lead to dementia. Psychiatric symptoms, such as anxiety, apathy, loss of self-esteem, and guilt are very frequent in the first stages of the disease, and often precede motor symptoms. The suicide rate is higher in patients with early onset or clinically presymptomatic individuals. Other frequent and disabling signs of HD are weight loss, sleep disturbances, and loss of circadian rhythm [119].

According to macroscopic and microscopic examinations, pathological changes in HD were classified by Vonsattel into four grades (0–4), each correlated with the degree of clinical impairment determined at the last pre-death evaluation [120].

Multiple processes seem to relate CAG expansion to neurodegeneration in HD, including transcriptional factors and coactivators, ultimately leading to cell death [108,121]. In HD, oxidative stress and mitochondrial defects are also revealed, the latter ones also leading to neuronal loss and reduced activity of the electron transport chain. HD is also accompanied by neurochemical alterations in dopamine, adenosine, and glutamate receptors [121,122,123,124].

Even today, the available pharmacological options for HD are only palliative [111,125,126,127].

**Figure 3:**
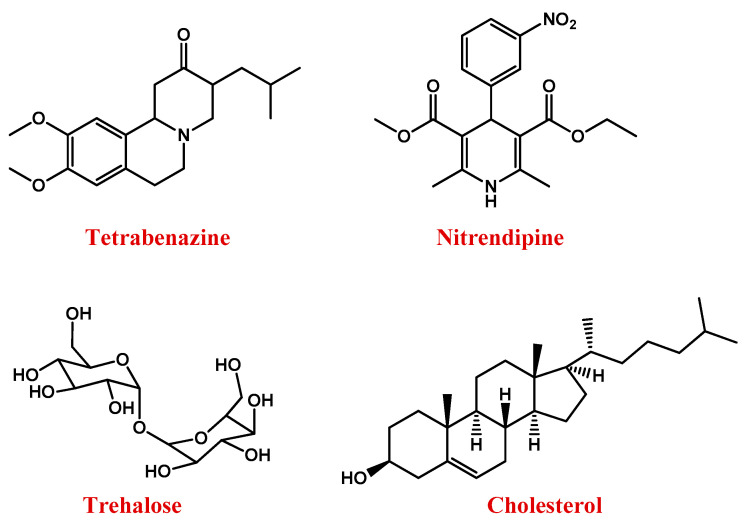
Molecular structures of main anti-HD drugs.

Antidopaminergic agents, e.g., tetrabenazine (TBZ, Figure 3), are the main drugs used for HD therapy [128,129]. Vesicular monoamine transporter 2 concentrates monoamines into vesicles, while TBZ reversibly inhibits this action, reducing presynaptic dopamine [128,130].

Nitrendipine (Figure 3) is a dihydropyridine calcium channel blocker that, exerting neuroprotective effects, can reduce the incidences of dementia in HD. However, being hydrophilic, it scarcely crosses the BBB [131].

Triggering a series of neuroprotective mechanisms, the disaccharide trehalose (Figure 3) can also improve cognitive performance in HD patients [132].

Several studies demonstrated that HD is also featured by abnormal brain cholesterol homeostasis [133,134]. Cholesterol dysregulation occurs in astrocytes [135,136] and is linked to the action of mHTT on sterol regulatory element-binding proteins (SREBPs) and its target genes, whose reduced transcription leads to lower production and a release of cholesterol in the brain. The low amount of cholesterol available to be taken up by neurons [137,138,139] impairs neuronal activities, causing brain malformations, and alterations in cognitive functions [140]. Therefore, strategies aimed at improving cholesterol delivery to neurons could be efficient in the treatment of the pathology [141,142].

On the other hand, neurotrophic factors are responsible for the growth, development, and survival of brain cells [143]. Changes in the levels and activities of neurotrophic factors, such as the brain-derived neurotrophic factor (BDNF), have been described in neurodegenerative disorders, including AD, PD, and HD [144,145,146,147]. Therefore, the use of genes that code BDNF can potentially prevent the death of brain cells and mitigate the symptoms of NDs [145,148].

**Figure 4:**
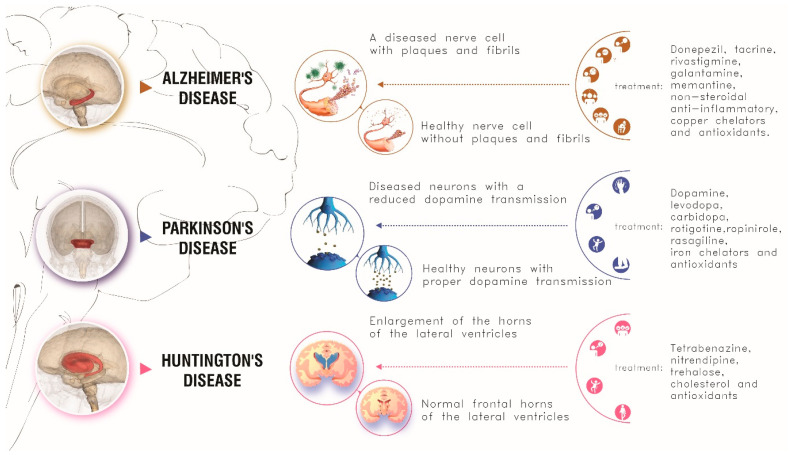
Main causes, symptoms and conventional treatments for the main three neurodegenerative disorders here described.

Causes, symptoms, and conventional treatments of these three neurodegenerative diseases are schematically summarized in Figure 4.

## 2. Oxidative Stress and Polyphenol Compounds in Neurodegenerative Diseases

For all the NDs above described, it has been reported that excessive oxidative stress is involved in neuronal damage [149]. Free radicals in the brain are formed as a consequence of oxidation processes or poor physiological antioxidant activity. In turn, ROS cause disruption in mitochondrial cellular lipids and proteins, as well as DNA damage, mitochondrial dysfunction, and genome instability [150,151,152,153].

Therefore, antioxidant agents have been widely investigated for their potential ability to prevent oxidative stress in NDs [154].

In this context, natural bioactive compounds, especially polyphenols, have gained increasing attention for their pharmacological and therapeutic potential [155,156,157,158,159].

A phenolic compound is a molecule having at least one aromatic ring on which one or more hydroxyl groups are attached. According to their main structural features, polyphenols can be classified as flavonoids and non-flavonoids with further subclasses [157,160,161].

**Figure 5:**
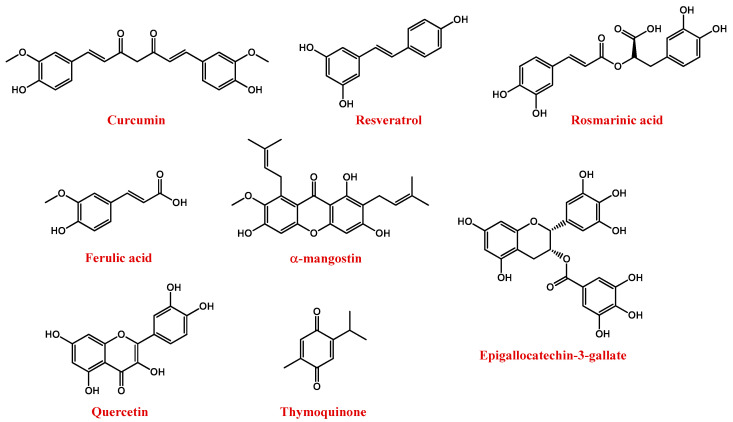
Molecular structures of polyphenol compounds here described.

Among polyphenols, the most important compounds are curcumin, resveratrol, rosmarinic acid, ferulic acid, α-mangostin, anthocyanins, epigallocatechin-3-gallate (EGCG), quercetin, and thymoquinone (Figure 5) [157].

Curcumin (Figure 5) is naturally present in turmeric (*Curcuma longa*), an important food and medication widely used in India and China. The chemical name of curcumin is diferuloylmethane and is a mixture of three major curcuminoids, i.e., curcumin, desmethoxy curcumin, and bis-desmethoxy curcumin [162].

Several studies proved that curcumin has antioxidant, anti-inflammatory, anticancer, antiviral, and antibacterial properties, thus exhibiting great potential in various diseases, including neurodegenerative ones [163,164,165]. Curcumin targets both β-amyloid and tau AD markers, decreasing the production of Aβ plaques, tau hyperphosphorylation and the formation of neurofibrillary tangles [166,167]. In the case of HD, curcumin showed beneficial effects, especially when added to the diet [168,169]. However, its poor bioavailability, resulting from its rapid metabolism and body clearance as well as poor BBB permeability, hindered its widespread use [170].

Resveratrol, or 3,5,4′-trihydroxystilbene (Figure 5), is a natural polyphenolic flavonoid and the main member of the stilbene family. It can be found in nature as both *cis* and *trans* isomers, the latter considered to be the most abundant and biologically active [171]. In particular, it is mainly present in the seeds and skins of grapes, red wine, mulberries, pomegranates, peanuts, tea, and rhubarb [172,173,174]. Resveratrol presents several effects, such as anti-cancer, anti-inflammatory, and anti-obesity [171]. The neuroprotective effects of resveratrol in NDs are related to its ability to reduce oxidative damage, toxicity, and apoptotic neuronal death [171,172,175].

Unfortunately, after intravenous injection, resveratrol is rapidly metabolized in the liver and intestine into both glucuronic acid and sulfate derivatives [176]. This results in a low resveratrol bioavailability which limits its pharmacological applications. In addition, resveratrol is also chemically unstable, since it is easily degraded by isomerization when exposed to elevated temperatures, pH changes, or UV light [171].

Rosmarinic acid (Figure 5) is a hydrophilic compound isolated from *Rosmarinus officinalis* L., which showed promising antioxidant properties linked to its ability to remove peroxynitrite anions and reduce inflammatory responses [177].

Ferulic acid (4-hydroxy-3-methoxycinnamic acid, Figure 5) exhibited both antioxidant and anti-inflammatory activities. In detail, it can reduce neuronal oxidative stress, preventing cell death [178,179].

α-mangostin (Figure 5) is a polyphenolic xanthone isolated from the pericarp, bark, and dried sap of *Garcinia mangostana* [180]. It exhibits a number of pharmacological effects, including neuroprotective, antioxidant, antitumour, and anti-neuroinflammatory actions [181,182,183,184].

Anthocyanins, polyphenolic compounds of the flavonoid family, have been found in fruits, grains, and flowers, and are reported to have antioxidant, anti-inflammatory, anti-apoptotic, and neuroprotective properties. Unfortunately, anthocyanins are chemically unstable, because their phenolic hydroxyl groups are easily oxidized to quinones with reduced biological activity [185,186,187].

EGCG (Figure 5), the major polyphenol in green tea, is known for its potent antioxidant properties [188]. In addition, it interacts with numerous proteins involved in NDs, such as Aβ amyloid, α-synuclein, and HTT [189,190,191].

Quercetin (Figure 5) is a dietary flavonoid with well-recognized antioxidant, anti-inflammatory and autophagy-inducer properties. Quercetin is present in apples, onions, parsley, berries, green tea, citrus fruits, and in some herbal remedies, e.g., ginkgo biloba [192]. For its potent neuroprotective action, quercetin represents a potential drug for the treatment of NDs [193,194,195].

Thymoquinone (2-isopropyl-5-methyl-1,2-benzoquinone, Figure 5) is the major active compound of the volatile oil of *Nigella sativa* seeds, featured by remarkable antioxidant, antitumour, anti-inflammatory, and immunomodulatory properties [196,197,198,199,200,201].

## 3. Targeting Brain: The Blood–Brain Barrier

Treatments of NDs are often clinically ineffective due to the poor accessibility of administered drugs to the desired site of action [202,203]. Indeed, delivering therapeutic agents to the CNS is one of the biggest challenges in biomedical approaches [204,205,206]. The main obstacle to the treatment of CNS diseases is represented by the BBB, which is a natural defence of the brain, being poorly permeable and highly selective to the passage of endogenous and exogenous substances, including drugs [207]. The BBB separates the blood from the extracerebral fluid and prevents the free diffusion of circulating molecules from the blood to the brain. BBB can exert this functionality thanks to its peculiar histological structure, which includes non-fenestrated endothelial cells connected by complex tight junctions, basement membranes, and astrocytic terminal feet [207,208,209,210]. Tight junctions almost completely seal the BBB and prevent access to the CNS of most compounds, especially large or hydrophilic molecules [211,212,213,214].

The absorption of compounds through the BBB mainly occurs through two different mechanisms, i.e., passive and active transport [205,215,216].

Passive transport or passive diffusion involves non-energetic transport pathways, such as paracellular and transcellular diffusion, exploited by hydrophilic and lipophilic compounds, respectively [217,218].

Paracellular diffusion: the interruption of the BBB due to local inflammation can lead to the weakening of the tight junctions, allowing the passage of polar compounds through the endothelial cells [219].

Transcellular diffusion: some small lipophilic and low molecular weight molecules (400–600 Da) can passively diffuse through the BBB [219].

Conversely, in the active transport, the movement of substances occurs through energy-dependent mechanisms, that allow overcoming biological gradients, such as concentration or electrochemical ones [216,220].

Carrier-mediated transport: small molecules such as ions, amino acids and glucose can be transferred from blood vessels to the extracellular space of the brain thanks to specific protein carriers [221]. In detail, glucose is transported through the glucose transporter 1 (GLUT-1) [222,223], while the L system is specific to the brain delivery of amino acids, such as valine, histidine, methionine, tyrosine and phenylalanine [224]. In turn, neutral amino acids, such as alanine, serine, and cysteine, can exploit the alanine/serine/cysteine transporter (ASC) [225].

Receptor-mediated transcytosis (RMT): large and/or hydrophilic molecules, like hormones and proteins, can be transported by specific receptors, expressed on the luminal side of endothelial cells in the brain parenchyma. First, the ligand binds its specific receptor, and then is easily internalized [226].

Adsorption-mediated transcytosis (AMT): this mechanism is mainly exploited by positively charged peptides or proteins and involves the endocytosis of the vesicles of the charged compound. It is a mechanism similar to RMT but is unspecific [219,226].

**Figure 6:**
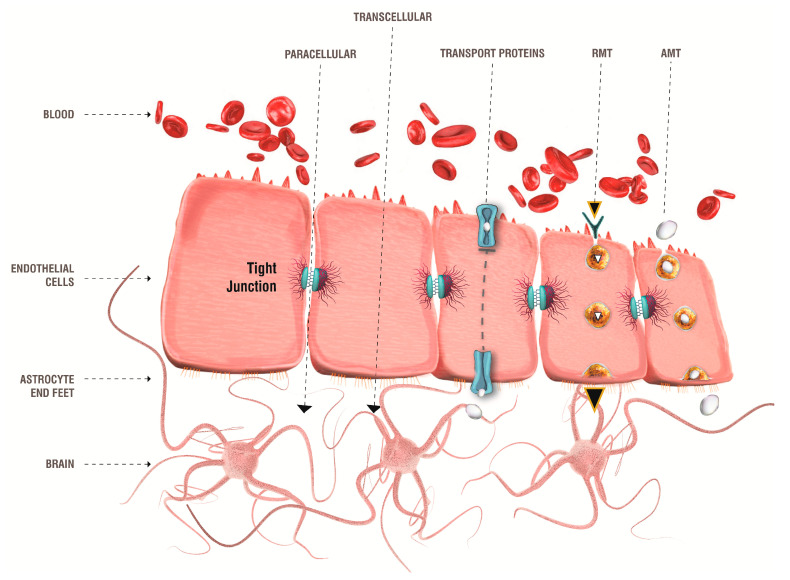
Schematic representation of the main methods for BBB crossing. AMT: adsorption-mediated transcytosis, RMT: receptor-mediated transcytosis.

Figure 6 illustrates the main transport systems through the BBB.

The lipophilicity and molecular weight are the main factors influencing the passive transport of substances through the brain endothelial cells. Although several drugs are inherently lipophilic, they are spontaneously and relatively easily reversed in the bloodstream. This is mainly due to the presence of highly effective efflux pumps, such as multi-specific organic anion transporter (MOAT), P-glycoprotein (Pgp) [227], and multidrug resistance-associated proteins (MRP) [228,229].

Thus, specific strategies have been developed to overcome the BBB and achieve efficient drug delivery to the CNS. These approaches include the biochemical opening, osmotic opening of tight junctions, or intra-cerebral implants. Generally, a hypertonic solution (containing mannitol) can be used to break the BBB. As an alternative, the interruption can be induced using angiotensin, bradykinin, or other similar agents. However, these approaches are very invasive, inducing long-term damage [3,230,231].

Considering the key role of lipophilicity in passive transport through the BBB, the chemical conversion of water-soluble molecules into fat-soluble compounds (e.g., through the covalent conjugation with lipids such as fatty acids or cholesterol [232]) has been widely investigated and generally pursued by adding lipophilic functional groups to mask the polar extremity of a selected drug and increase its chances of penetrating the BBB [3,230,233].

As an alternative approach, nanosystems of a different nature have been proposed as efficient targeted drug delivery systems for NDs [210,234,235,236,237,238,239,240,241,242].

## 4. Nanotechnology as a Tool to Reach the CNS in Neurodegenerative Diseases

To efficiently reach the CNS, nanotechnology offers suitable nanoplatforms on which the drug is loaded and then conveyed, favouring its selective release to the target site, reducing side effects and systemic exposure [210,234,235,236,237,238,239,240,241,242]. Nanomedicine provided a wide arsenal of versatile and multifunctional structures that can be applied for efficient brain drug delivery [234,235,236,237,238,239,240,241,242,243].

The term ‘nanoparticles’ (NPs) generally refers to materials with sizes below 200 nm in diameter showing higher cellular uptake than the larger microparticles, easily captured by Kupffer cells and eliminated by mononucleus phagocytes [244,245]. On the contrary, smaller NPs of less than 6 nm diameter are rapidly eliminated from the body by renal filtration [246,247].

The main advantages of nanomaterials are their size—comparable to those of biological macromolecules—their stability, and low-to-null systematic toxicity [243,248]. Nanosystems can also be finely tuned in their peculiar physico-chemical properties, such as shape, size, charge, hydrophobicity, and surface features [216,249,250]. Additionally, NPs exhibit a high surface area/volume ratio, which allows the functionalization of their surface—by covalent conjugation, encapsulation or adsorption [251]—with agents of different chemical nature, including suitable ligands for active targeting, hydrophilic polymers, and/or surfactants [250,252,253].

Targeting agents, able to recognize specific brain receptors or transporters, can improve drug delivery towards desired cells or organs and reduce the dosage necessary to obtain the therapeutic effect [254]. In addition, the functionalization with suitable targeting agents limits the unwanted NP accumulation in other organs or tissues of the body, such as the spleen, liver, and kidneys [218].

In this context, a large number of nanosystems were specifically designed to target transferrin (Tf) or lactoferrin (Lf) receptors, present at the endothelial cells of the BBB.

Transferrin receptor (TfR) is a transmembrane glycoprotein formed by two 90 kDa subunits connected by a disulfide bridge, each able to bind a transferrin molecule [255,256,257,258]. In turn, lactoferrin (Lf) is an 80 kDa naturally occurring iron-binding cationic glycoprotein belonging to the transferrin (Tf) family [259,260,261].

Additionally, cell-penetrating peptides (CPPs)—i.e., short peptides, typically consisting of less than 30 amino acids—have been deeply investigated as suitable carriers. They can indeed form complexes with a selected drug and facilitate its cell uptake, thanks to their intrinsic capability to translocate themselves into cells. Thus, CPPs can increase the bioavailability of a therapeutic agent of choice, favouring its intracellular or transcellular release, in a non-destructive way, and without significant toxicity [255,262,263,264].

To favour the efficient and selective brain release of drugs, NPs have also been functionalized with suitable nucleic acid-based aptamers, which are short oligonucleotides generally selected from a large random sequence pool by an iterative selection process, called Systematic Evolution of Ligands by EXponential enrichment (SELEX) [265,266,267]. Promising aptamers have been identified against several diseases [268,269,270,271,272,273,274,275,276,277], including neurodegenerative disorders, and thus explored as targeted ligands [278,279,280,281].

On the other hand, the surface modification with hydrophilic polymers makes NPs “invisible” to the immune system, thus increasing their in vivo half-life and reducing their immunogenicity. In fact, unmodified NPs tend to be opsonized and rapidly eliminated by the mononuclear phagocyte system. To overcome this problem, NPs can be coated with suitable polymers or surfactants, such as polyethylene oxide, polyethylene glycol (PEG) [282,283], tween 80 or polysorbate 80 (PS80), poloxamer [3], and poloxamine [218,244].

For example, PS80 can increase the chances of NPs to cross the BBB [284], probably after the adsorption of serum apolipoprotein E and/or B on the PS-coated NPs. Thus, NPs can mime low-density lipoproteins and interact with their receptors on the brain capillary endothelial cells [285].

However, the ability of NPs to cross the BBB is strongly dependent on the type, size, chemical surface features, and polarity of the particles. The exact mechanism by which nanomaterials can cross the BBB is not yet fully understood [286], even if endocytosis through the endothelial cells seems to be the most accredited process [3].

## 5. Nanosystems in the Treatment of Neurodegenerative Diseases

**Figure 7:**
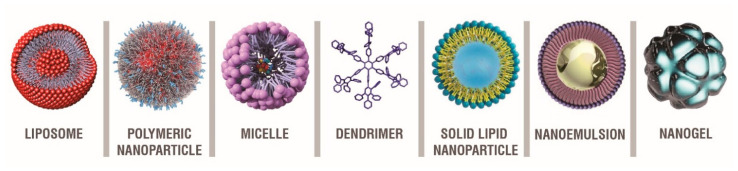
Main nanosystems exploited to delivery drugs to BBB in the treatment of neurodegenerative diseases.

Nanotechnology can greatly improve neuroprotection in NDs, and for this, several different nanoplatforms have been designed and studied as drug delivery systems (Figure 7) [242,287,288,289,290,291,292].

For example, polymeric NPs—i.e., solid polymeric, colloidal dispersion—have been extensively investigated to improve drug delivery across the BBB thanks to their well-proven biocompatibility, biodegradation, and no relevant toxicity. The most widely exploited polymers are poly(n-butyl cyanoacrylate) (PBCA), poly(lactic acid) (PLA) and poly(lactic-*co*-glycolic acid) (PLGA) [293,294,295,296].

PLGA, approved by the Food and Drug Administration (FDA), is one of the most successful biodegradable polymers, since it undergoes hydrolysis to produce lactic and glycolic acid, easily cleared from the body [297].

Moreover, chitosan, a natural and biocompatible polymer, demonstrated the ability to form polymeric NPs, which can effectively permeate the BBB, mostly due to their high positive charge density [298,299,300].

Solid lipid nanoparticles (SLNs) [301,302] and liposomes [303,304] also proved to be excellent carriers for the transport of potential anti-neurodegenerative drugs [305].

SLNs are essentially composed of an aqueous surfactant that includes dispersed melted lipids, and the drug is usually incorporated in the hydrophobic NP core. Remarkably, drug release from these NPs can be finely controlled and can last for months, due to their excellent drug entrapment ability and stability [306,307].

Liposomes are the most studied nanocarriers, consisting of vesicles made up of an aqueous core surrounded by a lipid bilayer, in which both hydrophilic and lipophilic drugs can be easily entrapped [308,309,310].

Cerasomes are biocompatible hybrid organic-inorganic liposomes with a liposome-like bilayer vesicular structure and a silicate surface [311].

In turn, spongosomes and cubosomes are liquid crystalline scaffolds formed by self-assembly of lyotropic lipids such as unsaturated monoglycerides, phospholipids and other co-lipids upon contact with an aqueous medium. Their structures involve bicontinuous lipid bilayers and networks of aqueous channels, allowing for the encapsulation of either hydrophilic or hydrophobic compounds [312,313].

In recent years, nanogels and nanoemulsions have attracted growing attention as ideal transport systems in NDs [314,315]. In particular, polymers forming crosslinked networks are able to combine ionic and non-ionic polymeric chains to form nanogels, which can carry oligonucleotides, proteins, low molecular weight drugs and other small molecules after swelling up in water [316,317]. In turn, nanoemulsions are oil-in-water formulations—consisting of an oil phase in dispersed droplets—especially suitable for the delivery of highly lipophilic drugs. They can also present surfactants and co-surfactants as additional ingredients to further stabilize the formulation [318].

Dendrimers are nanoscale-sized macromolecules with repetitive branching. The functional groups present at dendrimer extremities can be suitably modified to covalently link drug molecules and transport them into the brain [319].

When dispersed in a liquid phase above their critical micellar concentration, amphiphilic molecules—with a hydrophilic head and a hydrophobic tail—can self-aggregate to form micelles, that can be exploited as drug carriers [320].

Table 1 summarizes the main nanosystems used in the field of neurodegenerative diseases.

### 5.1. Alzheimer’s Disease

Several nanosystems have been developed to overcome the BBB and treat AD [321,322,323,324].

For example, Bhavna and colleagues proposed PLGA NPs coated with the non-ionic PS80 surfactant and functionalized with donepezil cholinesterase inhibitor [325]. These nanoformulations showed a mean particle size of ca. 90 nm in diameter, spherical shape, smooth morphology, and negative surface charge. Evaluated for 6 months, donepezil-loaded NPs proved to be stable at various temperatures and humidity conditions [325]. In vitro release studies indicated that donepezil exhibited a biphasic release pattern with an initial burst release (i.e., fast drug release, typically observed when the drug is on the NP surface), followed by a slower and continuous sustained release (generally due to the drug amount entrapped in the polymeric matrix). However, donepezil was completely released from the formulation within 25 days [325]. In order to study the in vivo biodistribution of the proposed formulations, ^99m^Tc-radiolabeled donepezil-loaded PLGA NPs and free radiolabeled donepezil solutions were also prepared. After intravenous administration, a higher percentage of radioactivity per gram was found in the mice brain in the case of the functionalized NPs compared to the radiolabeled drug solution [325].

In another study, Wilson et al. developed a similar system using PS80-coated PBCA NPs for the brain delivery of tacrine [326]. These NPs exhibited a mean size of less than 40 nm in diameter, negative Z-potential values and high stability, not changing their features during 3 months of monitoring. In vitro release studies proved a biphasic release pattern of the drug from the designed NPs. The initial burst effect occurred within 30 min and the remaining amount of tacrine was released in a sustained manner for 24 h [326]. After intravenous injection, a significantly higher tacrine concentration was found in the brain of treated Wistar rats, compared to the administration of the naked drug, while reduced amounts were detected in the spleen, lungs, and kidneys [326].

Carriers with a similar composition, i.e., PS80-coated PBCA, were also investigated for the brain delivery of rivastigmine [327]. Stable and almost spherical particles were thus obtained, featured by a mean size of ca. 50 nm in diameter and negative Z-potential values. In vitro release studies revealed a biphasic release pattern for rivastigmine from the NP formulation [327]. The concentration of rivastigmine in different organs taken from Wistar rats after intravenous injection of rivastigmine-loaded NPs was also evaluated and compared with that of the free drug. In the brain, a ca. 4-fold enhancement in the rivastigmine uptake was observed in the case of PS80-coated PBCA NPs compared to the bare drug [327].

In a subsequent investigation, rivastigmine was loaded on either PLGA or PBCA as polymeric carriers, obtaining NPs with average particle size of ca. 140 and 150 nm, respectively. In both cases, negative Z-potential values were found, especially for PLGA NPs [328]. For both formulations, pharmacodynamic studies demonstrated faster regain of memory in amnesic mice, compared to the free drug solution administration [328].

In a different design, rivastigmine was loaded in micelles composed of a PS80-coated amphiphilic copolymer. In detail, it comprised a hydrophilic α,β-poly(*N*-2-hydroxyethyl)-d,l-aspartamide (PHEA) backbone and a hydrophobic squalenyl-C17 (Sq17) portion linked by ethylenediamine (EDA) [329]. The so-called PHEA-EDA-Sq17-PS80 micelles showed a hydrodynamic diameter of ca. 30 nm, negative surface charge, and remained stable in human plasma up to 48 h [329]. In vitro biological assays evidenced no cytotoxic effects of either empty or loaded micelles on mouse neuronal Neuro2a cells. Moreover, the micelles were internalized by neuroblastoma cell lines, improving rivastigmine uptake, as compared with the free drug [329].

PLGA-based polymeric NPs were also used for the brain delivery of memantine [330]. In this work, to reduce the in vivo clearance of the NPs from the reticuloendothelial system (RES), their surface was further decorated with PEG [330]. NPs showed a mean size around 150 nm, negative surface charge, spherical shape, and smooth surface. Their features remained constant from 1 up to 6 months, depending on the temperature storage [330]. NPs were not cytotoxic on the tested brain cell lines (brain capillary endothelial bEnd.3 cells and astrocytes) and proved to cross the BBB, both in vitro and in vivo. Behaviour tests on transgenic APPswe/PS1dE9 mice proved that after oral administration, these formulations decreased memory impairment compared to the free drug [330]. Histological studies confirmed that these NPs attenuated β-amyloid plaques and the corresponding inflammation of AD [330].

Besides the agonists/antagonists of main neurotransmitters, also Aβ and tau aggregation inhibitors, such as antibodies, proteins, and peptides have been largely used for the decoration of different kinds of nanoplatforms in AD treatment [14,44,45,46,47].

Agyare and coworkers designed a small nanovehicle based on chitosan polymeric core, and coated it with the polyamine-modified F(ab’) portion of IgG4.1, an anti-amyloid antibody, able to target cerebral amyloids. For comparison, the authors also prepared a similar system coated with bovine serum albumin (BSA) as a control [331]. Both NPs—either functionalized with the antibody or BSA—showed ca. 200 nm diameter size and positive Z-potential values [331]. Antibody-decorated nanosystems showed the ability to cross the BBB and target the cerebrovascular amyloid in AD transgenic mice in a more efficient manner than the control nanovehicle [331].

Glutathione (GSH) is an endogenous tripeptide able to enhance the brain uptake of small chemical compounds [332]. GSH-PEG liposomes of ca. 110 nm size—based on either 1,2-dimyristoyl-*sn*-glycero-3-phosphocholine (DMPC) or egg yolk phosphatidylcholine (EYPC)—were prepared for the brain delivery of amyloid beta binding llama single-domain antibody fragments (VHH-pa2H) [333]. After administration via single intravenous bolus injection, both liposome formulations showed significantly reduced clearance profiles, as well as an increased amount of VHH-pa2H in perfused brains of APPswe/PS1dE9 double transgenic mice, compared to free VHH-pa2H [333].

Gobbi et al. demonstrated that anionic phospholipids such as phosphatidic acid (PA) and cardiolipin (CL) can serve as suitable Aβ targeting ligands [334]. Thus, for targeting β-amyloid plaques, they prepared both liposomes and SLNs, with a diameter of ca. 140 and 70 nm, respectively. Both systems showed negative Z-potential values and good stability over time [334].

Intrigued by these results, Re et al. developed bi-functionalized liposomes with a peptide derived from the apolipoprotein-E receptor-binding domain (mApoE), with the sequence CWGLRKLRKRLLR for BBB targeting, and with PA for Aβ binding [335,336]. Liposomes were composed of sphingomyelin (Sm) and cholesterol (Chol), and after functionalization with the selected peptides showed a mean diameter of ca. 120 nm [335,336]. The combination of the selected ligands on the liposome surface conferred them the ability of disaggregating Aβ assemblies in vitro. This ability, not displayed by monofunctionalized liposomes (with either PA or mApoE), arose from the synergic interaction of both the negatively charged PA phosphate group and the positively charged mApoE amino acids with oppositely charged residues present on Aβ peptide at physiological pH [336]. In addition, biocompatibility studies performed on different cell types (e.g., endothelial and neuroblastoma cells) indicated that liposomes did not affect the cell viability or the BBB monolayer integrity [336]. In vivo experiments demonstrated that the treatment with bifunctionalized liposomes induced a significant reduction in the number of brain plaques and remarkable recovery of Tg mouse impaired memory [335].

Zhang and colleagues developed PEG-coated PLA NPs decorated with two targeting peptides, i.e., TGNC and QSH [337]. TGNC (TGNYKALHPHNG) is a 12-amino acid long peptide able to target specific ligands at BBB [338], while the D-enantiomeric peptide QSHYRHISPAQV (denoted as QSH) has an excellent affinity to Aβ_1–42_ [339,340]. The obtained bis-functionalized NPs showed a mean diameter of 110 nm and negative Z-potential values [337]. In vitro cytotoxicity studies proved the safety of this system in both bEnd.3 and PC12 cells (derived from a pheochromocytoma of the rat adrenal medulla). The amount of NPs detected in the hippocampi of the AD mouse models was significantly higher by using the bis-functionalized NPs compared to naked NPs or NPs decorated with only one targeting peptide [337].

To efficiently deliver into the brain the iAβ5 peptide, previously developed by Soto et al. [48,49], Loureiro and colleagues investigated PLGA NPs suitably functionalized with two different mAbs as targeting ligands [341]. In detail, OX26 was selected for its well-known ability to recognize BBB cells expressing the transferrin receptor [342], while the anti-Aβ mAb DE2B4 was used to carry NPs directly to Aβ plaque deposits [341]. The functionalized PLGA NPs had a mean diameter around 150 nm, a spherical shape and negative surface charge. In vitro studies proved that the brain uptake of iAβ5-loaded NPs was substantially increased in the tested porcine brain capillary endothelial cells (PBCECs), compared to NPs without mAb functionalization [341].

Xiong et al. designed the Ac-LVFFARK-NH_2_ (LK7) peptide by incorporating two positively charged residues, R and K, into the central hydrophobic fragment of LVFFA, to improve electrostatic interactions with Aβ [343]. LK7 proved to inhibit Aβ fibrillogenesis in a dose-dependent manner, but it also showed high cytotoxicity toward human neuroblastoma SH-SY5Y cells, as a consequence of its strong self-assembly behaviour [343]. In order to limit toxicity issues, the designed LK7 peptide was then conjugated on PLGA NPs. These nanosystems exhibited a mean diameter of ca. 160 nm and negative Z-potential values. Compared to the bare peptide, these formulations showed markedly reduced cytotoxicity, however maintaining the ability to prevent Aβ fibrillogenesis [343].

In a different design, Chafekar et al. prepared branched dendrimers of 20–80 nm size for the delivery of the KLVFF peptide [344]. In detail, four KLVFF peptides were attached to the dendrimer (K4), and their effect on Aβ aggregation was compared to that produced by the monomeric peptide (K1) [344]. K4 proved to effectively inhibit Aβ aggregation in a concentration-dependent manner even more potently than K1. Moreover, K4 proved to disassemble existing aggregates. This study demonstrated the effectiveness of dendrimers as suitable scaffolds for neurodegenerative diseases in general, and AD in particular, and demonstrated the favourable impact of multivalency, i.e., the use of multiple copies of a selected therapeutic agent [344,345].

In a similar multivalency-based approach, Xiong et al. combined different peptides, able to inhibit the Aβ aggregation, in a single sequence, then attached to gold NPs [346]. The authors exploited portions derived from different Aβ regions, i.e., Soto’s β-sheet breaker peptide LPFFD [48,49] and the Aβ_39–42_ fragment VVIA [347]. In detail, CLPFFD (CLD6), CVVIA (CVA5) and two hybrid peptides—LPFFDCVVIA (LCA10) and VVIACLPFFD (VCD10) combining CLD6 and CVA5 in different ways—were conjugated onto AuNPs [346]. In all cases, functionalized NPs showed negative Z-potential values and size of ca. 15 nm in diameter. All the peptide-loaded nanosystems showed no toxicity on SH-SY5Y cells, along with improved ability to inhibit Aβ aggregation compared to each monovalent bare peptide [346].

Considering the multiple actions of vitamin D in different diseases [348,349], it is not surprising that vitamin d-binding protein (DBP) is involved in AD pathogenesis, being able to attenuate Aβ aggregation [350,351].

In this context, Jeon et al. prepared DPB-loaded PLGA NPs with an average particle size around 200 nm and an almost neutral surface charge [352]. These nanosystems significantly inhibited Aβ aggregation in vitro, not causing cytotoxicity on neuronal and glial cells (mouse hippocampal neuronal HT22 and human glioblastoma astrocytoma U373-MG cell lines). Moreover, these formulations proved to significantly attenuate Aβ accumulation, neuroinflammation, neuronal loss, and cognitive dysfunction in Aβ-overexpressing (5XFAD) mice [352].

Protoporphyrin IX has photosensitizer properties, thus can promote ROS production, which strongly suppresses Aβ aggregation [353]. On this basis, Xu and colleagues developed protoporphyrin IX-modified oxidized mesoporous carbon nanospheres (OMCNs) [354]. The 29-amino acid long brain-targeting peptide termed RVG (YTIWMPENPRPGTPCDIFTNSRGKRASNG) and derived from rabies virus glycoprotein, was also used as nanosphere decoration, being able to recognize *n*-acetylcholine receptors, widely expressed in the brain parenchyma cells and BBB [354]. The resulting functionalized OMCNs were positively charged with a size of approximately 110 nm. When dispersed in water, 10% FBS in DMEM or serum, these NPs showed only marginal changes in their size, suggesting very good stability up to 120 h [354]. These nanosystems showed no cytotoxicity on SH-SY5Y cells and efficiently inhibited tau phosphorylation and Aβ aggregation through ultrasound stimulation, also increasing the cognitive level of APP/PS1 transgenic mice [354].

In order to act on tau phosphorylation, Chen et al. proposed nanocrystals functionalized with methylene blue (MB), a tau aggregation inhibitor [355,356]. The nanocomposite was obtained assembling ultrasmall ceria nanocrystals (CeNCs) and iron oxide nanocrystals (IONCs) onto the surface of mesoporous silica nanoparticles (MSNs) [357]. Nanocrystal surface was further decorated with the T807 ligand with targeting ability for hyperphosphorylated tau [358]. The proposed nanosystem (indicated as CeNC/IONC/MSN-T807) showed a mean size of ca. 130 nm in diameter, positive Z-potential values and good stability, with no aggregation over a week in both water and cell culture medium [357]. MB-loaded CeNC/IONC/MSN-T807 not only possessed a high affinity to hyperphosphorylated tau, but also prevented some of the most important pathways of tau-associated AD pathogenesis. The authors proved that this system alleviated AD symptoms by mitigating mitochondrial oxidative stress, suppressing tau hyperphosphorylation, and protecting neuronal death, both in vitro and in vivo [357].

A different approach for AD treatment is based on the use of anti-inflammatory or antioxidant agents, most of which was derived from natural sources.

In this context, Amin and coworkers prepared osmotin-loaded dextran-coated Fe_3_O_4_ magnetic nanoparticles (MNPs) [359]. In detail, Fe_3_O_4_ MNPs were chosen for their superparamagnetic properties associated with high biocompatibility [360], while dextran coating was selected, since it enhances the blood circulation time and further stabilizes NPs [361] Osmotin-loaded MNPs showed spherical shape and an average diameter of 90 nm [359]. In vitro cytotoxicity studies revealed that MNPs did not possess any significant cytotoxic effects on the tested SH-SY5Y cell lines [359]. Using an electromagnetic function to guide MNPs into brain sites, the authors found that MNPs were able to reduce Aβ accumulation, beta-site amyloid precursor protein cleaving enzyme 1 (BACE-1) expression, synaptotoxicity, memory impairment, and tau hyperphosphorylation in a Aβ_1–42_-treated mouse model, in comparison with native osmotin [359].

In order to improve the brain permeability of d-penicillamine, NPs based on either 1,2 dioleoyl-*sn*-glycero-3-phosphoethanolamine-*N*-(4-[p-maleimidophenyl]-butyramide) (sodium salt) (MPB-PE) or 1,2-dioleoyl-*sn*-glycero-3-phosphoethanolamine- *N*-(3-[2-pyridyldithio]-propionate) (sodium salt) (PDP-PE) were covalently linked to the copper chelator by disulfide or thioether bonds, which can be easily broken with reducing agents to release the active compound. d-penicillamine/NP conjugates showed ca. 110 nm size, and did not show any tendency to aggregate over time [362]. After treatment under reducing conditions, such as dithiothreitol, NPs effectively resolubilized copper-Aβ_1–42_ aggregates [362].

To improve the poor bioavailability of tarenflurbil (or R-flurbiprofen), Mu and colleagues proposed its encapsulation on PEG–PLA NPs. The resulting nanosystems were further decorated with an RNA-based aptamer, FB4 (5′-NH_2_-spacer-CGCGGAUUGCGGCCGUUGUCUGUGGCGUCCGUUC-3′), proved to specifically bind to the extracellular domain of the mouse transferrin receptor [363]. Functionalized NPs showed a mean diameter of ca. 25 nm and negative Z-potential values. The resulting nanoformulations greatly increased the solubility of tarenflurbil in aqueous solution and improved its controlled release in vitro. Furthermore, these formulations significantly enhanced the delivery efficacy of tarenflurbil into bEND.5 cells, proving the unique targeting ability of the FB4 aptamer [364].

In a recent investigation, Arora et al. prepared liposomes for the targeted delivery of the *BDNF* gene. To this aim, liposomes—mainly composed of dioleoyl-3-trimethylammonium propane chloride (DOTAP), dioleoyl-*sn*-glycero-3-phosphoethanolamine (DOPE), Chol and 1,2-distearoyl-*sn*-glycero-3-phosphoethanolamine (DSPE) PEG—were functionalized with mannose, as GLUT-1 targeting ligand and penetratin or rabies virus glycoprotein as CPPs. All the targeting ligands were covalently bound to DSPE-PEG lipid chains [365]. In detail, plasmid encoding BDNF (pBDNF) was first complexed with chitosan through electrostatic interactions, and then this complex was loaded into liposomes [365]. Liposome formulations exhibited an average size of less than 200 nm and a slightly positive charge. Surface-modified liposomes proved to successfully protect encapsulated genes against enzymatic degradation and showed significantly improved transfection of *BDNF* in primary astrocytes and neurons, compared to unfunctionalized liposomes. In addition, these nanosystems proved to cross the BBB following a single intravenous administration in C57BL/6 mice without causing inflammation or toxicity [365].

Thanks to their antioxidant and neuroprotective properties, a large number of polyphenol compounds have been used to functionalize different NPs for neurodegenerative disorders [156,157,158].

For example, to overcome the poor bioavailability of curcumin (Cur), several nanosystems, especially polymeric NPs and liposomes, have been developed for its effective delivery through the BBB [366,367,368,369,370].

Shaikh et al. reported Cur-loaded PLGA NPs with particle size of 264 nm and spherical shape [371]. In vivo pharmacokinetic studies proved that these formulations increased the bioavailability of curcumin by at least 9-fold, compared with bare Cur [371].

Moreover, Tiwari and colleagues studied Cur-loaded PLGA NPs, demonstrating their ability to potently induce neural stem cell proliferation and neuronal differentiation in vitro and in hippocampus and subventricular zone of adult rats [372].

In vivo studies from Khalil et al. proved that, compared with Cur alone, both PLGA and PEG-coated PLGA NPs effectively improved ca. 16- and 55-fold the bioavailability of Cur, decreasing its metabolism and sustaining its delivery [373]. For both formulations, particles were smaller than 200 nm in diameter, and the encapsulation efficiency was found to be over 70% [373].

Fascinated by the intriguing properties of these systems, different research groups improved curcumin-loaded polymeric NPs using various targeting agents of peptide [374,375,376,377] or oligonucleotide nature [378].

For example, Mulik et al. prepared PS80-coated PBCA-based NPs for Cur delivery and further decorated this system with the isoform of ApoE3 [374], whose complexation prevents the transport of Aβ across BBB [379]. Both Cur-PBCA and ApoE3-Cur-PBCA formulations exhibited negative Z-potential values and a mean particle size of ca. 180 and 200 nm, respectively. The use of ApoE3 clearly increased the uptake of curcumin into the SH-SY5Y cells; hence, the enhanced antiapoptotic activity of curcumin was observed, compared to Cur solution or nontargeted NPs [374].

In contrast, Mathew and colleagues used the Tet-1 peptide (HLNILSTLWKYR), previously identified by Park et al. [380], as BBB targeting ligand [375]. This is a 12-amino acid peptide with binding characteristics similar to tetanus toxin [380,381]. The synthesized NPs showed a diameter in the 150–200 nm range, smooth surface and negative Z-potential values [375]. These nanosystems maintained the antioxidant and anti-amyloid properties of Cur, while the functionalization with Tet-1 greatly increased in vitro neuronal targeting efficiency of NPs in GI-1 glioma cells compared to free Cur [375].

Vandelli et al. modified Cur-loaded PLGA NPs with the BBB crossing GFTGFLS-O-β-d-glucose-CONH_2_ glycopeptide, known as g7 [382,383]. These NPs showed a mean diameter around 200–250 nm and negative Z-potential values [376]. Functionalized nanosystems did not show toxicity on the tested hippocampal cells, but proved to reduce oxidative stress, decrease Aβ aggregation, and promote Aβ disaggregation more efficiently than free Cur [376].

In a similar design, Fan et al. explored the B6 peptide (CGHKAKGPRK) targeting TfR [377]. The mean diameter of the PEG-PLGA-B6 NPs was less than 100 nm without Cur, and slightly increased to 150 nm after Cur encapsulation [377]. In vitro assays demonstrated good blood compatibility and increased cellular uptake for NPs compared to free Cur. Ex vivo analysis proved that the designed formulations were able to reduce the hippocampal β-amyloid formation and tau hyperphosphorylation. In vivo tests on APP/PS1 transgenic mice showed the attenuation of memory loss and cognitive impairment, compared with native Cur [377].

In another study, Mathew and colleagues explored the RNA-based aptamer named NN2 (5′-UGCCACUCUCCUGGGACCCCCCGCCGGAUGGCCACAUCC-3′) as a targeting agent, previously identified by SELEX procedure against amyloid β-peptide [384]. In detail, this aptamer was covalently attached on the surface of Cur-loaded PLGA NPs, obtaining final formulations with a mean particle size of ca. 170 nm [378]. In vitro, NPs did not show significant cytotoxicity on LAG cell line (mouse fibroblast-like connective tissue) and proved to effectively reduce the size of amyloid aggregates [378].

Furthermore, liposomes were widely investigated as drug delivery systems for curcumin [366,367,368,369,370].

Lazar et al. described nanoliposomes in which Cur was covalently attached to 1,2-dipalmitoyl-*sn*-glycero-3-phosphothioethanol (sodium salt) (DPSH) via Michael addition obtaining DPS–Cur [385]. Then, liposomes were prepared by mixing 1,2-dipalmitoyl-*sn*-glycerol-3-phosphatidylcholine (DPPC) and Chol with 20% in mol of DPS–curcumin. Final formulations showed a mean diameter of ca. 200 nm and negative surface charge, also proving to be stable for more than 1 month [385]. They were non-toxic in vitro on human SH-SY5Y cells, downregulated the secretion of amyloid peptide and partially prevented Aβ-induced toxicity. Additionally, liposomes strongly bound Aβ deposits in post-mortem brain tissue of APPxPS1 mice and AD patients [385].

As polymeric NPs, liposomes have also been decorated with suitable targeting ligands to improve the BBB permeability, and thus the bioavailability of a selected therapeutic agent of choice.

In particular, Taylor et al. prepared Cur-functionalized nanoliposomes, in which Cur was either incorporated in the lipid bilayer of liposomes or used as surface decoration covalently bound via click-chemistry reactions [386]. In the first case, liposomes were composed of a matrix of Sm/Chol mixed or not with 5 or 20 mol% of PA, CL or GM1 ganglioside [386], known as lipids able to target Aβ_1–42_ aggregates [334]. In the case of the click-chemistry reaction, liposomes were composed of DPPC, 1,2-distearoyl-*sn*-glycerol-3-phosphatidylcholine (DSPC), cholesterol and a polyethylene glycol lipid (Y, lipid-polyethylene glycol-N3-3-deoxy-1,2-dipalmitoyl-3-(4′-methyl (O-(2-azidoethyl)-heptaethylenglycol-2-yl)-ethylcarbamoylmethoxy ethylcarbamoyl-1H-1′,2′,3′-triazol-1′-yl)-*sn*-glycerol) in the DPPC/DPPG/Chol/Y 8:2:10:1 or 2 molar ratio [386].

Depending on the specific strategy used for Cur functionalization and the chosen targeted lipid, liposomes showed a mean diameter in the 100–200 nm range and negative Z-potential values. All the prepared formulations proved to inhibit the formation of Aβ_1–42_ fibrils, especially in the case of the covalent attachment of Cur on the liposome surface [386].

Mourtas and coworkers prepared multifunctional liposomes, incorporating lipid-PEG-Cur derivatives (DPS-PEG_2000_-Cur), and further decorated with the anti-Tf mAb OX26 [387]. The obtained nanoformulations showed a mean diameter lower than 160 nm and a negative surface charge. OX26 decoration significantly improved the brain intake in the human brain capillary endothelial hCMEC/D3 cells. Notably, both untargeted and targeted Cur-loaded liposomes showed a high affinity for amyloid deposits, as detected on post-mortem brain samples of AD patients [387].

In a different approach, Hagl and coworkers prepared curcumin micelles containing 93% of PS80 and 7% of Cur [388]. After micelle administration, curcumin bioavailability was examined, both in vitro in PC12 cells and in vivo in NMRI mice [388]. The use of micelles sensibly improved the bioavailability of Cur around 10- to 40-fold in plasma and brain of mice, respectively [388].

Using a different nanosystem, Cur-functionalized silica-coated Au NPs featured by hydrodynamic diameter of 10–25 nm were also investigated. Cur was covalently linked on the NP surface, determined as strongly positive [389]. These nanosystems proved to inhibit amyloid fibrillation in a dose-dependent manner and were able to dissolve preformed amyloid fibrils [389].

In a very intriguing design, Meng and coworkers proposed low-density lipoprotein (LDL)-mimic nanostructured lipid carrier (NLC) loaded with Cur and modified with Lf to improve the brain-targeted delivery of the native polyphenol [390]. LDL is a 22–27 nm diameter nanoparticle, composed of a core of hydrophobic lipids, primarily cholesteryl esters and triacylglycerols, and showing phospholipids, unesterified cholesterol and a single molecule of apolipoprotein B 100 (ApoB-100), as stabilizing agents [391]. Being a natural molecule, LDL is non-immunogenic and long-circulating. In addition, the overexpression of low-density lipoprotein receptors (LDLR) in brain capillary endothelial cells (BCECs) is expected to improve brain targeting [392].

In this work, NLC was composed of PC, Chol oleate and glycerol trioleate—mimicking the composition of the lipid portion of LDL—and Lf was adsorbed on the NLC surface via electrostatic interactions [390]. Functionalized NLCs showed a mean diameter of ca. 100 nm and negative Z-potential values [390]. Compared to untargeted NPs, Lf-functionalized nanosystems showed a ca. 1.4-fold enhanced uptake in the selected BCECs cell line associated with a sustained release of the active moiety. Remarkably, fluorescence resonance energy transfer (FRET) studies proved that Cur remained inside NLC after cell uptake [390]. Lf-decorated NPs effectively permeated the BBB, preferentially accumulating in the brain with an efficiency ca. 3-fold higher than untargeted NPs, as revealed by ex vivo imaging studies [390].

In another approach, Kuo and Tsai proposed the co-encapsulation of curcumin and rosmarinic acid onto polyacrylamide (PAAM)-CL-PLGA NPs [393] PAAM is a synthetic cationic biopolymer with well-known biocompatibility [394], while CL is a phospholipid able to bind Aβ with high efficacy [334]. The authors further decorated this system with the surface 83–14 mAb [393], able to favour brain crossing via receptor-mediated endocytosis [395]. These formulations showed spherical geometry and an average diameter lower than 100 nm [393]. These NPs did not induce relevant toxicity to human brain microvascular endothelial cells (HBMECs). Experimental evidence revealed that an increase in the concentration of the mAb enhanced the permeability of the drugs through the BBB in human neuroblastoma SK-N-MC cells. In turn, at a fixed antibody concentration, Cur showed higher permeability than rosmarinic acid [393].

Among natural compounds exploited as therapeutics for AD, resveratrol has also aroused growing interest due to its neuroprotective properties [176,396].

Loureiro and colleagues demonstrated that extracts of the grape seed and grape skin strongly inhibit Aβ_1–42_ fibril formation, showing improved effect compared to pure resveratrol [397]. For the brain delivery of grape skin and seed extracts, the authors explored SLNs with a lipid phase composed of cetyl palmitate, and PS80 as a stabilizer [397]. These NPs were also functionalized with OX26 [342]. All the formulations showed a mean diameter between 170 and 190 nm, with almost neutral Z-potential values, and they were stable for at least one month [397]. Experiments on human brain-like endothelial cells showed that the cellular uptake of the OX26-decorated SLNs was substantially more efficient than that of untargeted SLNs and SLNs functionalized with an unspecific antibody [397].

The same research group also prepared resveratrol-loaded SLNs with a different covalently attached targeting agent, i.e., ApoE [398]. The resulting NPs showed spherical shape, particle size lower than 200 nm in diameter, and negative Z-potential values. They were also stable up to 6 months [398]. In vitro cytotoxic effects were assessed in the hCMEC/D3 cell line and revealed no significant toxicity. The permeability through hCMEC/D3 monolayers showed a ca. 2-fold increase for ApoE-functionalized resveratrol-loaded NPs with respect to untargeted SLNs [398].

To improve ferulic acid delivery, Picone and colleagues investigated SLNs with a mean diameter of less than 100 nm and a highly negative surface charge [399]. Ferulic acid treatment, especially after encapsulation into SLNs, decreased ROS generation, restored mitochondrial membrane potential and reduced cytochrome c release, and intrinsic pathway apoptosis activation [399].

Chen et al. prepared α-mangostin-loaded liposomes carrying Tf covalently attached on the liposome surface [400]. These nanosystems showed a mean diameter lower than 200 nm and a negative surface charge [400]. In vitro studies in bEnd.3 cells demonstrated the ability of these liposomes to cross the BBB as integrated systems. The evaluation of the in vivo brain distribution indicated that Tf-modified liposomes significantly improved the brain delivery of α-mangostin [400].

Kim et al. used antioxidant anthocyanins for the decoration of PEG-coated AuNPs [401]. The mean size of the conjugated NPs was found to be around 130 nm, accompanied by negative Z-potential values [401]. In vitro bioactivity assays indicated no relevant variations in the viability of normal HT22 hippocampal neuronal cells after NP treatment. A clear accumulation of the anthocyanin-modified PEG-AuNPs was evidenced in the BV2 microglial cells, as well as in mice brains [401]. The authors demonstrated that both free anthocyanins and those conjugated onto NPs reduced Aβ_1–42_-induced neuroinflammatory and neuroapoptotic markers by inhibiting the p-JNK/NF-κB/p-GSK3β pathway in both in vitro and in vivo AD models. However, anthocyanin-loaded NPs were more effective compared to free anthocyanins [401].

Similarly, Amin et al. prepared spherically shaped anthocyanin-loaded PEG-PLGA NPs [402]. The average particle size of these nanosystems was around 165 nm in diameter with negative Z-potential values. In vitro drug release studies demonstrated a biphasic release profile of the anthocyanin, reaching a complete release within 3 h [402]. Cytotoxicity investigations in SH-SY5Y cells proved that NPs were not cytotoxic on the tested cell line. In addition, compared to free anthocyanin, anthocyanin-conjugated NPs exhibited more potent neuroprotective properties. In particular, these formulations effectively attenuated AD’s markers like APP, BACE-1, neuroinflammatory markers such as p-NF-kB (phospho-nuclear factor kappa B), TNF-α (tumour necrosis factor α) and iNOS (inducible nitric oxide synthase), and neuroapoptotic markers, including Bax, Bcl2 and Caspase-3 protein expressions [402].

Considering the low delivery efficiency of EGCG, Zhang and colleagues prepared Tet-1 peptide-coated EGCG-stabilized selenium NPs with an average diameter in the 25–29 nm range [403]. These formulations effectively inhibited Aβ fibrillation and disaggregated preformed Aβ fibrils into nontoxic aggregates. In addition, both targeted and untargeted NPs bound Aβ fibrils with a high affinity and Tet-1 peptide significantly enhanced the cellular uptake of these systems in PC12 cells [403]. Moreover, in vitro studies demonstrated that these NPs were able to protect PC12 cells from Aβ-induced damage by suppressing the generation of ROS and DNA fragmentation [403].

Cano et al. proposed the combination of EGCG and ascorbic acid on PEG-PLGA NPs, resulting in an average size around 120 nm and a negative surface charge [404]. Oral administration of these formulations in a APPswe/PS1dE9 AD mice model provided high EGCG accumulation in brain. Although for both, free ECGC and drug-loaded NPs initial EGCG amounts were similar, pharmacokinetic studies proved that long-term (5–25 h) concentrations were ca. 5-fold higher with dual-drug loaded NPs [404]. In addition, NP treatment markedly reduced neuroinflammation and Aβ plaque enhancing spatial learning and memory [404].

The combination of two different drugs on the same platform was also explored by Kuo and colleagues, who prepared quercetin and rosmarinic acid-loaded liposomes, composed of DPPC, Chol and dihexadecyl phosphate (DHDP) [405]. In all cases, liposomes showed an average diameter lower than 160 nm and a net negative surface charge. Immunochemical experiments in HBMEC cells proved that the designed multifunctional liposomes were able to penetrate the BBB [405]. In Sprague–Dawley male rats, these liposomes decreased acetylcholinesterase activity, lipid peroxidation level, and Aβ plaque formation [405].

### 5.2. Parkinson’s Disease

Also in the case of PD several nanosystems have been proposed as effective tools to deliver therapeutics to the CNS [406,407,408].

Lopalco and colleagues encapsulated dopamine into liposomes composed of soy phosphatidylcholine (PC), Chol and DSPE-PEG_2000_-COOH, functionalized or not with Tf conjugated to the carboxyl group of DSPE-PEG chains [409]. Unfunctionalized liposomes exhibited a mean diameter of ca. 160 nm, while functionalized nanosystems showed a larger size, with a diameter of around 180 nm. The charge of both formulations was found to be slightly positive, and no significant variation in terms of size was detected after 1 month [409]. In vitro transport of formulations across the BBB was investigated using human hCMEC/D3 cell monolayers. Permeability studies revealed that functionalized liposomes were able to cross the cell membrane monolayer by exploiting a receptor-mediated endocytosis mechanism, with remarkably higher permeability compared with the unfunctionalized systems [409].

In a different approach, dopamine-loaded chitosan NPs—with 110–150 nm particle size and positive Z-potential values—were prepared [410]. These NPs proved to reduce the overall toxicity of dopamine in Madin–Darby canine kidney (MDCKII-MDR1) cells. In addition, dopamine-loaded chitosan NPs increased the transport of dopamine in the selected cell line and reduced ROS production [410]. After intraperitoneal acute administration, dopamine-loaded NPs induced a dose-dependent increase in striatal dopamine output consistent with a prompt and pulsate release of the neurotransmitter, as demonstrated by brain microdialysis experiments in rat [410].

In the context of polymeric NPs, Pahuja and colleagues investigated dopamine-loaded PLGA nanosystems featured by a slow and constant release of the neurotransmitter [411]. The dopamine-containing NPs had hydrodynamic diameter of around 120 nm, and slightly negative Z-potential values [411]. Treatment of SH-SY5Y cells with these nanoformulations did not cause a reduction in cell viability or morphological changes, as compared to cells treated with free dopamine. These NPs were also able to cross the BBB and capillary endothelium in the striatum and substantia nigra in a 6-hydroxydopamine (6-OHDA)-induced rat model of PD [411]. After intravenous NP administration, significantly increased dopamine levels and reduced dopamine-D2 receptor supersensitivity were found in the striatum of parkinsonian rats. Furthermore, these NPs significantly reversed neurobehavioural deficit in the tested animal models. Notably, dopamine delivery through nanoformulations did not cause alterations in the heart rate and blood pressure, not evidencing any pathological changes in the brain or other peripheral organs [411].

More recently, Jahansooz and colleagues developed dopamine-loaded PBCA NPs with mean size of 100 nm and negative surface charge [412]. These NPs were able to cross the BBB, providing a sustained release of dopamine. As a consequence, their administration improved brain function in a 6-OHDA-induced PD animal model (Wistar rats), particularly reducing α-synucleinopathy and reversing behavioural abnormalities [412].

Rashed et al. delivered dopamine across the BBB using a nanoparticle system consisting of polyvinylpyrrolidone-poly(acrylic acid) (PVP/PAAc) nanogels [413]. The hydrodynamic mean diameter of free nanogels and dopamine-loaded nanogels was found to be about 600 nm in both cases [413]. The functionalized nanogels were then intraperitoneally administered at different doses and dosing regimens in PD models of Wistar rats (reserpine- and rotenone-induced), and the catalepsy score and striatal dopamine levels were evaluated. The administration of dopamine-loaded nanogels induced a significant improvement in catalepsy state, accompanied by a significant increase in dopamine content in the rat striatum [413].

Chlorotoxin (ClTx), originally isolated from scorpion venom of *Leiurus quinquestriatus*, is a 36-amino acid long peptide [414], which was exploited by Xiang and colleagues for the targeted drug delivery of l-DOPA-decorated liposomes [415]. In detail, liposomes were composed of hydrogenated soy phosphatidylcholine (HSPC), Chol and DSPE-PEG in HSPC/Chol/DSPE-PEG 20:10:2 molar ratio, and ClTx was covalently attached to DSPE-PEG chains. Both unmodified and ClTX-modified liposomes showed an average particle size around 100 nm with slightly positive Z-potential values [415]. ClTx modification highly improved the uptake of liposomes by brain microvascular endothelial cells in vitro. In mice treated through intraperitoneal injections, the active targeting system significantly improved the distribution of both dopamine and dihydroxyphenyl acetic acid (DOPAC), metabolites of l-DOPA, in the substantia nigra and striata in comparison with untargeted liposomes or free l-DOPA. In 1-methyl-4-phenyl-1,2,3,6-tetrahydro pyridine (MPTP)-induced C57 mice PD model, these liposomes significantly attenuated behavioural disorders [415].

A dopamine agonist, i.e., rotigotine, was loaded onto PLGA-based microspheres [416]. In an MPTP-induced PD mouse model, rotigotine-loaded microspheres, more effectively than free drug, improved the Parkinsonism score, protected dopaminergic neurons, reduced the microglial cell activation and release of neuroinflammatory cytokines [416].

The dopamine agonist ropinirole (RP) was loaded onto uncoated and PS80-coated chitosan NPs, obtaining particle sizes of ca. 200 nm [417]. Both formulations exhibited a biphasic release pattern with an initial burst release of RP, followed by a sustained release over 10 h. Coated NPs were found stable over 3 months [417]. After 1 h from intravenous administration in Wistar rats, higher RP concentrations were found in the brain, with less accumulation in the liver, spleen and kidney for coated NPs, as compared to uncoated NPs and free RP. Additionally, NP-induced hemolytic tests were performed on PS80-coated chitosan NPs, fully demonstrating their hemocompatibility [417].

More recently, ropinirole was encapsulated into SLNs, nanostructured lipid carriers (NLCs), and their corresponding hydrogel (RP-SLN-C and RP-NLC-C) formulations [418]. RP-SLN and RP-NLC systems showed particle size around 200 nm, negative surface charge, and proved to be stable over 3 months. These NPs were then converted to hydrogels using carbopol 934 as a gelling polymer [418]. In vitro and ex vivo permeation studies showed sustained release profiles and enhanced permeation compared with the suspension of free RP, used as control [418]. Pharmacokinetics studies showed a 2.1 and 2.7-fold enhancement for RP-SLN and RP-NLC oral administration, 3.0 and 3.3-fold enhancement for RP-SLN-C and RP-NLC-C topical administration compared to suspensions of free RP, respectively. Furthermore, RP-SLN-C and RP-NLC-C showed a 1.4 and 1.2-fold bioavailability enhancement after topical administration compared to the oral one [418]. After treatment, pharmacodynamic investigations in Wistar rats proved the restoration of biochemical changes with enhanced dopamine, glutathione, and catalase levels, as well as reduced lipid peroxidation levels [418].

The antiparkinsonian drug bromocriptine was incorporated in two different nanoformulations, i.e., monoolein aqueous dispersions (MADs) and NLCs. Both MADs and NLCs showed a mean diameter lower than 200 nm [419]. Compared to MAD-based formulations, NLCs proved to markedly attenuate motor deficit in 6-OHDA hemilesioned rats, providing long-lasting therapeutic effects with respect to free bromocriptine [419]. Thus, although both formulations were able to efficiently encapsulate the dopamine agonist, only NLCs extended its half-life in vivo [419].

Hu et al. used PEG-PLGA NPs as carrier for urocortin (UCN) and Lf functionalization to improve its brain delivery [420]. Targeted UCN-NPs were endowed with a mean diameter of ca. 120 nm and a negative surface charge [420]. Intravenous injection of UCN-loaded Lf-NPs effectively attenuated the striatum lesion in 6-OHDA in rats [420].

In the contest of iron chelation therapy, Wang and colleagues selected non-Fe hemin (NFH) as a natural prototype iron chelator, obtained through the removal of the iron core of hemin [421]. Then, they prepared NFH-loaded NPs composed of acrylated BSA coated with the zwitterionic poly(2-methacryloyloxyethyl phosphorylcholine) (PMPC) [421]. As an additional ingredient of NP formulations, the authors explored the covalent conjugation on the NP surface of the HIV-1 trans-activating transcriptor (TAT) CPP—with the sequence GRKKRRQRRRPP-OCH_3_—as BBB permeable shuttle [421]. These formulations showed less than 30 nm diameter and slightly negative Z-potential values [421]. Additionally, these NPs did not show toxicity on SH-SY5Y cells, even at high NFH concentrations. The final system proved to efficiently accumulate in the brain—where it remained for even 48 h—producing significative neuroprotective effects both in vitro and in vivo. In particular, it was able to reverse functional deficits in MPTP-induced PD mice, providing both physiological and behavioural improvements [421].

In a different approach, nanomicellar formulations of the antioxidant coenzyme Q10 (CoQ10) were developed [422]. Nanoformulations were composed of CoQ10 and polyoxyethanyl-α-tocopheryl sebacate (PTS) in 1:2 molar ratio. PTS is an amphiphilic molecule, possessing both hydrophilic (PEG-600) and lipophilic (α-tocopherol) portions, separated by an aliphatic spacer of sebacic acid, and it has self-emulsifying properties. The studied system exhibited a mean diameter of ca. 20 nm [422]. The use of micelles effectively improved the brain permeation of CoQ10 after oral administration and blocked the ongoing neurodegeneration in MPTP-treated mice. However, if the treatment was withdrawn, the neurodegeneration resumed, indicating that these nanosystems were not able to reverse the damage initiated by MPTP death pathway [422].

Chen and coworkers successfully formulated schisantherin A, a promising natural antioxidant compound, in nanocrystals, with an average diameter of ca. 160 nm. These formulations showed a fast dissolution rate in vitro, but the drug was quickly eliminated in vivo after oral administration [423]. To overcome this problem, schisantherin A was thus encapsulated in methoxy PEG–PLGA NPs, obtaining systems with a mean diameter of ca. 70 nm and negative Z-potential values, which remained essentially unchanged after 1 week [424]. These nanosystems demonstrated extended drug circulation in the bloodstream, and consequently an increased brain uptake compared to the free compound. Additionally, these systems showed stronger neuroprotective effects in zebrafish and cell culture models of PD [424]. In zebrafish, these NPs gradually dissociated, providing a sustained drug release over time. Notably, they were still intact in the intestine and brain 2 h after the administration [424].

A high number of curcumin-loaded formulations have been investigated in vitro and in vivo for PD treatment, also in combination with other natural products of biological interest or targeting agents [366,367,368,369,370].

For example, neuroprotective effects of curcumin against rotenone-induced neurotoxicity were confirmed in vitro when encapsulated onto Lf-modified NPs [425]. These formulations exhibited ca. 60 nm diameter size and a negative surface charge. Functionalized NPs showed enhanced intracellular drug uptake, sustained retention, and improved neuroprotection as compared to the bare drug. In detail, these nanosystems reduced ROS levels and the expression of α-synuclein in the tested neuroblastoma SK-N-SH cells [425].

Zhang and colleagues prepared Cur-encapsulated PS80-modified cerasomes with a mean diameter of about 110 nm and negative surface charge [426]. Surface modification of cerasomes with PS80 surfactant proved to effectively enhance the penetration of Cur-loaded cerasomes across the BBB. In addition, these nanoformulations exhibited a longer circulation lifetime than free Cur, due to an initial burst release of the drug followed by its sustained delivery [426]. In addition, with the assistance of the ultrasound-targeted microbubble destruction (UTMD) technique to induce the BBB opening regionally and non-invasively, localized delivery of the functionalized NPs into the mouse brain was achieved [426]. Notably, after C57BL/6 mice treatment, motor behaviour and dopamine levels were restored to normal expression [426].

In another study, the efficacy of Cur-loaded alginate-based NPs was evaluated in a transgenic drosophila model of PD [427]. After treatment, a significant dose-dependent delay in the loss of climbing ability and reduction in oxidative stress, lipid peroxidation, and apoptosis in the brain of flies was observed [427].

Kundu and colleagues proposed the co-encapsulation of curcumin and piperine—an alkaloid from the pepper plant *Piper nigrum* with anti-apoptotic and anti-inflammatory effects [428,429]—in a single platform consisting of glyceryl monooleate NPs coated with various surfactants. The final system showed a mean hydrodynamic diameter lower than 100 nm and a negative surface charge [430]. In vitro release kinetics studies suggested a sustained release of both drugs from NPs for 8 days, with a typical biphasic pattern [430]. These formulations inhibited the aggregation of α-synuclein protein into oligomers and fibrils in vitro and reduced rotenone-induced cell death in PC12 cells via decreasing oxidative stress and apoptosis, and simultaneously enhancing autophagic activity [430]. Further in vivo studies demonstrated that these NPs were able to cross the BBB ameliorating motor coordination [430].

In a different study, Rakotoarisoa and colleagues studied the neuroprotective roles of monoolein-based spongosome and cubosome NPs loaded with Cur and fish oil, rich in omega-3 polyunsaturated fatty acids [431].

These bifunctionalized nanocarriers showed a bimodal distribution, indicating the coexistence of large size (∼400 nm) particles (cubosomes or spongosomes) and small (∼100 nm) vesicles or precursors of intermediate-type liquid crystalline structures [431]. In addition, they did not show cytotoxic effects on SHSY5Y cells, and significantly attenuated ROS accumulation in these cells as compared to the aqueous suspension containing bare drugs [431].

Da Rocha Lindner et al. prepared resveratrol-loaded PS80-coated PLA NPs, featured by a mean size of ca. 200 nm in diameter and a negative surface charge [432]. The neuroprotective effects of resveratrol-loaded NPs in C57BL/6 mice confirmed higher neuroprotection against MPTP-induced neurochemical and behavioural changes as compared to free resveratrol administration [432]. Remarkably, NP administration also protected against MPTP-induced striatal tyrosine hydroxylase loss and lipid peroxidation [432].

### 5.3. Huntington’s Disease

In the context of HD treatment, Manjunath and Venkateswarlu prepared SLNs loaded with nitrendipine, and their pharmacokinetics and bioavailability were assessed after intravenous and intraduodenal administration to male Wistar rats [433]. SLNs were composed of various triglycerides (trimyristin, tripalmitin and tristearin), soy PC 95%, poloxamer 188, and charge modifiers (dicetyl phosphate and stearylamine). The average size of SLNs with different lipids, with and without charge modifiers, was in the 100–120 nm range. Z-potential values were found to be highly negative in the absence of charge modifiers or in the presence of the dicetyl phosphate as lipid. On the contrary, the use of stearylamine gave, in all cases, a net positive surface charge [433]. Compared to the nitrendipine suspension, which presented an elevated drug concentration in the brain over 3 h, nitrendipine-loaded SLNs showed a high drug amount for at least 6 h. In particular, after intraduodenal administration, positively charged SLNs improved the bioavailability of nitrendipine from 3- to 5-fold, independently from the specific triglyceride used [433]. When compared with nitrendipine suspension, SLNs were better taken up by brain and moderately taken up by heart, liver, and spleen after intravenous administration. Negatively and positively charged SLNs were better taken up by liver and brain, respectively [433].

Within a different approach, Debnath and colleagues designed poly(trehalose) NPs, composed of a 6 nm iron oxide core and 10−15 nm polyacrylate shell containing covalently linked trehalose [434]. The zwitterionic shell allowed high cell uptake without cytotoxicity in a mutant mouse neuroblastoma HD150Q cell line [434]. These NPs resulted in being 1000−10,000 times more efficient than molecular trehalose in inhibiting protein fibrillation, in blocking aggregation of polyglutamine-containing mHTT in model neuronal cells, and in suppressing mHTT aggregates in HD mouse brain. Compared with free trehalose, which was effective only beyond millimolar concentration, nanoformulations proved to be efficient also at micromolar concentrations [434].

Exploring the multivalency effect, the incorporation of trehalose onto NPs (ca. 80–200 molecules per NP) proved to be crucial for efficient brain targeting, effective entry into neuronal cells, and suppression of mHTT aggregation [434].

Aiming at obtaining increased cholesterol delivery into the brain, Valenza and coworkers investigated biodegradable and biocompatible polymeric PLGA NPs modified with the g7 glycopeptide [382,383] and loaded with cholesterol [435]. These NPs showed average diameter lower than 200 nm and negative surface charge [435]. In contrast to unmodified NPs, g7-targeted systems demonstrated efficiently crossing BBB and localizing in glial and neuronal cells in different brain regions within a few hours after systemic injection. On the other hand, repeated systemic intraperitoneal injections in a transgenic mouse model of HD (R6/2) also proved to rescue synaptic and cognitive impairments in the tested animals [435].

In a different work, Sandhir et al. evaluated the neuroprotective efficacy of Cur-loaded SLNs against 3-nitropropionic acid (3-NP)-induced HD [436]. Moreover, 3-NP is indeed a neurotoxin that replicates the neurodegenerative phenotype of HD, being able to induce the loss of mitochondrial function by selectively inhibiting the activity of Complex II of the respiratory chain, thus increasing ROS production in striatal neurons from HD transgenic mice [437].

SLNs were composed of stearic acid as a solid core, lecithin as surfactant, and taurocholate as co-surfactant, and showed an average diameter in the 70–150 nm range [436]. Cur nanosystems proved to markedly increase the activity of brain mitochondrial complexes and cytochrome levels in the treated animals. Cur-loaded SLNs also restored the glutathione levels and superoxide dismutase activity. Moreover, after this treatment, a significant reduction in mitochondrial swelling, lipid peroxidation, protein carbonyls, and ROS formation was observed in rats [436]. In addition, treated animals showed significant improvements in neuromotor coordination compared with control rats [436].

SLNs with similar composition were also proposed for the brain delivery of thymoquinone [438]. These formulations showed spherical shape, particle size around 170 nm in diameter, and negative Z-potential values [438]. Then, thymoquinone-encapsulated SLNs were evaluated in vivo for their influence against 3-NP induced HD model in comparison with the free thymoquinone suspension. These nanosystems significantly improved body weight, also ameliorating the histopathological alterations of the tested animals. Furthermore, NPs administration proved to improve muscle strength, rigidity, movement, and memory performances compared to the untreated group [438]. In subsequent studies, these nanoformulations proved to suppress microglial activation, NMDA receptor stimulation, and neuroinflammation [439].

Debnath et al. described quercetin nanoformulations composed of polyaspartic acid-based polymer micelles, with a mean diameter in the 35−70 nm range [440]. Quercetin-loaded nanosystems proved to enter into HD150Q cells via endocytosis, where they slowly released the drug in more than 3 days. In addition, this system showed anti-amyloidogenic properties at lower quercetin concentration (1 μM vs. 20−100 μM range necessary for quercetin alone), and inhibited polyglutamine aggregation in the HD cell model [440]. Compared to the native quercetin, these formulations also provided more efficient autophagy induction and better anti-amyloidogenic performance at a lower dose [440].

In a different approach, Mandal and colleagues designed PL-based NPs end-conjugated with the anti-amyloidogenic trehalose or the neurotransmitter dopamine or arginine. The final system was also loaded with catechin, an anti-amyloidogenic polyphenolic compound [441]. These formulations showed a hydrodynamic diameter of around 200 nm and Z-potential values close to zero [441]. The designed NPs proved to cross the neuronal cells, where they inhibited polyQ aggregation and reduced oxidative stress [441].

A useful approach in HD treatment is based on the use of peptides able to inhibit polyglutamine aggregation [442].

In this frame, Joshi and co-workers explored PLGA-based polymeric NPs for the efficient brain delivery of the PGQ_9_[P^2^] peptide (KKQQQQQQQQQPGQQQQPQQQQPGQ QQQQQQQQPGQQQQQQQQQKK). The authors obtained spherical NPs with mean diameter lower than 200 nm and negative surface charge [443]. In vitro release kinetics demonstrated that NPs released PGQ_9_[P^2^] via erosion and diffusion processes. When PGQ_9_[P^2^]-loaded NPs were incubated with the aggregation-prone Q_35_P_10_ peptide, representing the N-terminal part of HTT protein, inhibition of Q_35_P_10_ elongation and aggregation was observed [443].

Other effective strategies for HD treatment are based on gene silencing, especially on short-interfering RNA (siRNA), aiming at decreasing the expression of mHTT [114].

Godinho et al. loaded a previously developed siRNA [444]—composed from 5′-GCCUUCGAGUCCCUCAAGUCC-3′ as sense strand and 5′-ACUUGAGGGACUCGAAGGCCU-3′ as antisense strand—in NPs composed by the assembly of modified amphiphilic cationic β-cyclodextrin (β-CD) molecules [445]. Indeed, modified β-CDs—based on naturally occurring oligosaccharide molecules—are promising oligosaccharide-based carriers that bind siRNAs, strongly protecting them from enzymatic degradation [446]. In particular, positively charged modified β-CDs are thought to strongly interact with negatively charged siRNAs via electrostatic interactions, as found for other cationic delivery systems [447,448,449,450,451,452]. These nanosystems showed a hydrodynamic diameter between 100 and 350 nm and a net positive surface charge. In addition, they were stable at 37 °C in artificial cerebrospinal fluid up to 6 h, therefore adequately protecting siRNA from degradation [445]. Moreover, these complexes were able to reduce the expression of the *HTT* gene in rat striatal cells (ST14A-HTT120Q) and human HD primary fibroblasts. After NP treatment, only limited toxicity was observed in all the in vitro models tested [445]. Sustained knockdown effects were observed in the striatum of the R6/2 mouse HD model after a single direct NP injection. In contrast, repeated brain injections resulted in a selective alleviation of motor deficit [445].

**Table 1:** Nanosystems for therapeutic applications in neurodegenerative diseases.

Disease	Nanocarrier Platform	Composition	Bioactive Agent	Active Targeting Ligand	Ref.
Alzheimer	Polymeric NPs	PS80-coated PLGA NPs	Donepezil, cholinesterase inhibitor		[325]
	Polymeric NPs	PS80-coated PBCA NPs	Tacrine, cholinesterase inhibitor		[326]
	Polymeric NPs	PS80-coated PBCA NPs	Rivastigmine, cholinesterase inhibitor		[327]
	Polymeric NPs	PLGA and PBCA NPs	Rivastigmine, cholinesterase inhibitor		[328]
	Micelles	PHEA-EDA-Sq17-PS80 amphiphilic copolymer	Rivastigmine, cholinesterase inhibitor		[329]
	Polymeric NPs	PEG-PLGA NPs	Memantine, glutamate antagonist		[330]
	Polymeric NPs	Chitosan	F(ab’) portion of the anti-amyloid antibody IgG4.1		[331]
	Liposomes	PEG-DMPC and PEG-EYPC	Amyloid beta binding llama single-domain antibody fragments (VHH-pa2H)	GSH	[333]
	Liposomes	Sm/Chol in 1:1 molar ratio		PA and CL	[334]
	SLNs	Stearic acid (internal phase), phospholipon 90G (surfactant) and sodium taurocholate (co-surfactant)		PA and CL	[334]
	Liposomes	Sm/Chol in 1:1 molar ratio		PA and mApoE peptide	[335,336]
	Polymeric NPs	PEG-PLA NPs		TGN and QSH peptides	[337]
	Polymeric NPs	PLGA NPs with pluronicF127 (0.1%) as stabilizer	iAβ5 peptide, Aβ aggregation inhibitor	Anti-TfR mAb OX26 and anti-Aβ mAb DE2B4	[341]
	Polymeric NPs	PLGA NPs	Ac-LVFFARK-NH_2_, Aβ aggregation inhibitor		[343]
	Dendrimers		KLVFF peptide, Aβ aggregation inhibitor		[344]
	Gold NPs		LCA10 and VCD10 peptides, Aβ aggregation inhibitors		[346]
	Polymeric NPs	PLGA NPs	Vitamin d-binding protein		[352]
	Nanospheres	Oxidized mesoporous carbon nanospheres	Protoporphyrin IX, Aβ and tau aggregation inhibitor	RVG peptide	[354]
	Nanocrystals	CeNC/IONC/MSN-T807	Methylene blue, tau aggregation inhibitor	T807 ligand	[357]
	Magnetic NPs	Dextran coated-Fe_3_O_4_ NPs	Osmotin protein, neuroprotective		[359]
	Liposomes	MPB-PE or PDP-PE	d-penicillamine, copper chelator		[362]
	Polymeric micelles	PEG-PLA	R-flurbiprofen (or tarenflurbil), anti-inflammatory	FBA, RNA aptamer	[364]
	Liposomes	DOTAP/DOPE/Chol/DSPE-PEG (4.5:4.5:2:4 molar ratio)	BDNF	Mannose and penetratin or rabies virus glycoprotein	[365]
	Polymeric NPs	PLGA NPs	Curcumin		[371,372]
	Polymeric NPs	PLGA and PEG-PLGA NPs	Curcumin		[373]
	Polymeric NPs	PS80-coated PBCA NPs	Curcumin	ApoE3 peptide	[374]
	Polymeric NPs	PLGA NPs	Curcumin	Tet-1 peptide	[375]
	Polymeric NPs	PLGA NPs	Curcumin	g7 glycopeptide	[376]
	Polymeric NPs	PEG-PLGA NPs	Curcumin	B6 peptide	[377]
	Polymeric NPs	PLGA NPs	Curcumin	NN2, RNA aptamer	[378]
	Liposomes	DPPC/Chol (2:1 molar ratio) with DPS–curcumin (20 mol %)	Curcumin		[385]
	Liposomes	Sm/Chol in 1:1 molar ratio	Curcumin	PA or CL or GM1 ganglioside	[386]
	Liposomes	DPPC/DPPG/Chol/Y (8:2:10:1 or 2 molar ratio)	Curcumin		[386]
	Liposomes	DSPC/Chol (2:1 molar ratio) with DPS-PEG_2000_-Cur (10 or 20 mol %)	Curcumin	Anti-TfR mAb OX26	[387]
	Micelles	PS80	Curcumin		[388]
	Gold NPs	Silica-coated Au NPs	Curcumin		[389]
	NLCs	PC/Chol oleate/glycerol trioleate (1:0.06:0.21 molar ratio)	Curcumin	Lactoferrin	[390]
	Polymeric NPs	PAAM-CL-PLGA NPs	Curcumin and rosmarinic acid	83–14 mAb	[393]
	SLNs	Cetyl palmitate and PS80 as stabilizer	Resveratrol, grape skin and seed extracts	Anti-TfR mAb OX26	[397]
	SLNs	Cetyl palmitate and PS80 as stabilizer	Resveratrol	ApoE peptide	[398]
	SLNs	Compritol 888 ATO (lipid matrix), Epikuron 200 (surfactant) and sodium taurocholate (co-surfactant)	Ferulic acid		[399]
	Liposomes	DSPC/Chol/DSPE-PEG2000/DSPE-PEG_2000_-COOH (2:1:0.11:0.021 molar ratio)	α-mangostin	Transferrin	[400]
	Gold NPs	PEG-coated AuNP_S_	Anthocyanins		[401]
	Polymeric NPs	PEG-PLGA NPs	Anthocyanins		[402]
	Selenium NPs		EGCG	Tet-1 peptide	[403]
	Polymeric NPs	PEG-PLGA NPs	EGCG and ascorbic acid		[404]
	Liposomes	DPPC/Chol/DHDP (5:4:1 molar ratio)	Quercetin and rosmarinic acid	ApoE peptide and PA	[405]
Parkinson	Liposomes	PC/Chol (7:3 molar ratio) with DSPE-PEG_2000_-COOH (2.5 mol %)	Dopamine	Transferrin	[409]
	Polymeric NPs	Chitosan	Dopamine		[410]
	Polymeric NPs	PLGA NPs	Dopamine		[411]
	Polymeric NPs	PBCA NPs and poloxamer 188 as stabilizer	Dopamine		[412]
	Nanogels	PVP/PAAc	Dopamine		[413]
	Liposomes	HSPC/Chol/DSPE-PEG 20:10:2 molar ratio	l-DOPA, dopamine precursor	Chlorotoxin peptide	[415]
	Microspheres	PLGA NPs	Rotigotine, dopamine agonist		[416]
	Polymeric NPs	PS80-coated chitosan	Ropinirole, dopamine agonist		[417]
	SLNs	Dynasan-114 (solid lipid), soylecithin (primary surfactant) and poloxamer 188 (secondary surfactant)	Ropinirole, dopamine agonist		[418]
	NLCs	Dynasan-114 (solid lipid), Caproyl 90 (liquid lipid) soylecithin (primary surfactant) and poloxamer 188 (secondary surfactant)	Ropinirole, dopamine agonist		[418]
	MADs	Glyceryl monooleate and poloxamer 407	Bromocriptine, dopamine agonist		[419]
	NLCs	Tristearin/Miglyol 2:1 molar ratio with poloxamer	Bromocriptine, dopamine agonist		[419]
	Polymeric NPs	PLGA and PEG-PLGA NPs	Urocortin	Lactoferrin	[420]
	Zwitterionic polimers	PMPC-coated acrylated BSA	Non-Fe hemin, iron chelator	TAT peptide	[421]
	Micelles	PTS	Coenzyme Q10, antioxidant		[422]
	Nanocrystals	Pluronic F68	schisantherin A, antioxidant		[423]
	Polymeric NPs	mPEG–PLGA NPs	schisantherin A, antioxidant		[424]
	Micelles		Curcumin	Lactoferrin	[425]
	Cerasomes	PS80-modified cerasome-forming lipid *N*-[*N*-(3-triethoxysilyl)propylsuccinamoyl]-di-hexadecylamine	Curcumin		[426]
	Polymeric NPs	Sodium alginate	Curcumin		[427]
	Liposomes	Glyceryl monooleate NPs coated with Pluronic F-68 and vitamin E−TPGS	Curcumin and piperine		[430]
	Spongosomes and cubosomes	Monoolein	Curcumin and fish oil		[431]
	Polymeric NPs	PS80-coated PLA NPs	Resveratrol		[432]
Huntington	SLNs	Triglycerides (trimyristin, tripalmitin and tristearin), soy PC 95%, poloxamer 188 and charge modifiers (dicetyl phosphate and stearylamine)	Nitrendipine		[433]
	Magnetic NPs	Iron oxide core and switterionic polyacrylate shell	Trehalose		[434]
	Polymeric NPs	PLGA NPs	Cholesterol	g7 glycopeptide	[435]
	SLNs	Stearic acid (solid core), lecithin (surfactant) and taurocholate (co-surfactant)	Curcumin		[436]
	SLNs	Stearic acid (solid core), lecithin (surfactant) and taurocholate (co-surfactant)	Thymoquinone		[438,439]
	Polymeric micelles	Polyaspartic acid	Quercetin		[440]
	Polymeric NPs	PL NPs modified with trehalose/dopamine/arginine	Catechin		[441]
	Polymeric NPs	PLGA NPs	PGQ9 peptide		[443]
	Oligosaccharide-based NPs	β-cyclodextrin	siRNA		[445]

## 6. Nanosystems for Nose-to-Brain Drug Delivery in the Treatment of Neurodegenerative Diseases

The nasal cavity exerts a fundamental protective function of filtering, warming, and humidifying the inhaled air before it reaches the lower airways. Nasal administration of drugs using the nose-to-brain pathway allows for direct drug targeting in the brain, bypassing the BBB and avoiding the first-pass effect [453,454].

Indeed, through the nasal route, a selected drug reaches the brain directly by using the trigeminal and olfactory nerves, located in the upper part of the nose. In this way, problems such as low water solubility, poor bioavailability or enzymatic degradation are completely avoided. Furthermore, intranasal administration has been shown to deliver drugs into the CNS at a higher rate and extent than other conventional administration routes [455,456,457].

Other advantages of intranasal administration are represented by the possibility of self-administration, improved patient compliance, rapid onset of action, minimized systemic exposure, and reduced potential peripheral side effects [458].

Several nasally administered formulations proved their efficacy for delivering drugs to the brain in NDs [459,460,461,462,463,464]. The nanosystems mainly used are nanoemulsions [465,466] and liposomes [467,468], but also polymeric NPs [469] and SLNs [470].

To improve drug absorption in the nasal mucosa, these nanosystems have usually been functionalized with lectins, proteins or glycoproteins, able to specifically recognize sugar molecules, binding the glycosylated membrane components of nasal mucosa, as well as improving the bioadhesivity of the delivery system [471,472].

Table 2 summarizes the main nanosystems for nose-to-brain drug delivery in the treatment of neurodegenerative diseases.

### 6.1. Alzheimer’s Disease

For AD treatment, donepezil was encapsulated in chitosan nanosuspensions with an average size of ca. 150–200 nm [473]. The developed nanosuspensions were intranasally instilled into the nostrils of Sprague–Dawley rats with the help of cannula. Compared to free drug administration, the use of formulations sensibly enhanced both brain and plasma concentration. In vivo studies also proved that no mortality, haematological or body weight changes, or toxicity occurred in animals after administration demonstrating an overall good tolerability of these formulations [473].

In a different design, donepezil was loaded into liposomes—composed of DSPC/Chol/PEG—showing ca. 100 nm size in diameter and negative surface charge. After intranasal administration of liposomal formulation in Wistar rat, the bioavailability of donepezil in plasma and brain significantly increased compared to other organs [474].

Liposomes were also used as drug delivery system for another cholinesterase inhibitor, i.e., tacrine. In detail, liposomes were prepared with cholesterol and EYPC, partly enriched with α-tocopherol and/or omega-3 fatty acids [475]. All the liposome formulations showed a mean diameter in the 175–219 nm range, with negative Z-potential values and excellent encapsulation efficiency [475]. Along with good mucoadhesive properties, these multifunctional liposomes showed a marked increase of tacrine permeability across phospholipid vesicle-based barriers and sheep nasal mucosa. The use of α-tocopherol in the formulation proved to further increase the neuroprotective activity and antioxidant properties of tacrine-functionalized liposomes [475].

Nasal delivery of tacrine was also investigated by using BSA NPs carrying β-CD or its hydrophilic derivatives (hydroxypropyl β-CD and sulfobutylether β-CD). These NPs presented a mean diameter lower than 260 nm, spherical shape and negative surface charge [476]. These formulations showed in vitro and ex vivo good mucoadhesion properties on sheep nasal mucosa [476].

Yang and co-workers investigated CPP-modified liposomes for the nasal delivery of rivastigmine [477]. The authors first studied the difference in the plasma rivastigmine concentration at 15, 60 and 240 min after intravenous or intranasal administration. When intravenously administered, rivastigmine showed the lowest level in the plasma, probably due to rapid kidney uptake. In contrast, intranasal rivastigmine administration provided a higher plasma concentration of the drug [477]. Liposomes were prepared using EPC, Chol and DSPE-PEG lipids, at which the CPP of sequence GLPRRRRRRRRR was covalently attached. These formulations showed a mean diameter of ca. 170 nm and a negative surface charge [477]. After administration, they provided enhanced permeability of rivastigmine across the BBB by murine brain microvascular endothelial cells. These liposomes effectively improved rivastigmine distribution and its retention in CNS regions, especially in the olfactory bulb, hippocampus and cortex, compared to free drug [477].

Haider and colleagues prepared rivastigmine nanoemulsions for enhanced brain delivery through nasal administration [478]. For the preparation of nanoemulsions, Capmul MCM was selected as an oil component, while PS80 and transcutol P were chosen as surfactant and co-surfactant, respectively. Nanoemulsions were spherically shaped, negatively charged, and with a mean diameter of ca. 30 nm in size [478]. Ex vivo diffusion studies on goat nasal mucosa proved that the drug permeation was higher when administered as nanoemulsion than as a free drug solution. Moreover, in vivo studies demonstrated that the brain concentration of rivastigmine reached with the nanoemulsions was significantly higher than that obtained with the pure, non-formulated cholinesterase inhibitor [478]. Moreover, nasal ciliotoxicity studies of the goat nasal mucosa confirmed the presence of a normal intact epithelial layer with no nasociliary damage, demonstrating that the components of this nanoemulsion were safe, non-toxic and non-irritating to the nasal mucosa [478].

In a further design, rivastigmine was intranasally delivered by chitosan-based NPs [479]. These formulations showed a mean particle size lower than 200 nm and a positive surface charge. In vitro release studies indicated a controlled and sustained release profile of rivastigmine from NPs [479]. Brain concentration achieved after intranasal administration of chitosan NPs in Wistar rats was significantly higher than that achieved after intravenous or intranasal administration of free rivastigmine solution [479].

Li and co-workers encapsulated galantamine in liposomes composed of soy PC and Chol, obtaining spherical particles with an average diameter of ca. 110 nm and highly negative Z-potential values [480]. Brain and plasma concentration of the drug—determined after 4 days administration of liposome formulations—were significantly higher than those of free drug solution [480]. Moreover, liposomes did not show relevant toxicity on PC-12 cells, and notably, the cytotoxicity of galantamine was clearly reduced when it was loaded in liposomes [480]. Acetylcholinesterase activity in the brain, determined after 10-day treatment using rat brain homogenates, was higher when liposomes were intranasally administered. In contrast, less efficacy was found for intranasal and then oral administration of free galantamine [480].

Tarenflurbil was loaded into two different nanocarriers, i.e., PLGA NPs and SLNs, for intranasal administration [481]. Both formulations showed ca. 200 nm diameter size, negative surface charge and high drug entrapment efficiency. Moreover, NPs proved to ensure the transcellular transport of the drug across olfactory axons [481]. Improved brain biodistribution profiles of tarenflurbil were obtained for both nanosystems, as compared with the free drug solution. Among NPs, polymeric ones showed comparatively better efficacy in delivering tarenflurbil to the brain as evidenced by pharmacokinetic parameters in the brain and blood [481].

In the study by Gao et al., the vasoactive intestinal peptide (VIP), a 28-amino acid neuroprotective peptide [482], was efficiently incorporated into the PEG-PLA NPs modified with wheat germ agglutinin (WGA) [483]. In detail, WGA from *Triticum vulgare* specifically bound N-acetyl-D-glucosamine and sialic acid, both of them abundantly found in the nasal cavity [484]. VIP-functionalized NPs exhibited a spherical shape and an average diameter of ca. 90–100. A slight increase in size was observed after WGA conjugation, reaching 100–120 nm range in diameter [483]. VIP was efficiently entrapped in the selected nanosystems via electrostatic interactions established between cationic VIP and negative-charge-carried PLA fragment of the polymer. In vitro studies demonstrated that the incorporation of VIP into PEG-PLA NPs strongly enhanced its stability, both in the nasal wash and in plasma [483]. In vivo data suggested that the modification of WGA on the surface of NPs significantly increased the amount of intact VIP delivered to CNS, and improved the effect of VIP-loaded-NPs on the impairment in the rats treated with ethylcholine aziridium (AF64A), especially alleviating the reduction of acetylcholinesterase activity in the hippocampus of AF64A-treated rats [483].

In a different work, Zheng and co-workers investigated the effectiveness of nasal delivery of amyloid protein breaker H102 peptide, with the sequence HKQLPFFEED, as a liposome formulation [485]. These liposomes were composed of EPC, DSPE-PEG_2000_ and Chol in 20:1:5 molar ratio. Their mean particle size was ca. 110 nm and their surface charge was found to be slightly negative [485]. When intravenously administered, H102 was undetectable in plasma after 5 min, in accordance with its short plasma half-life of ca. 2 min. In contrast, when intranasally administered, both H102 solution and liposomes were detected at the same time point. The comparison of H102 solution with its liposome formulation indicated a slower systemic absorption and higher drug concentration in the plasma due to liposome encapsulation [485]. When intravenously administrated, H102 was undetectable in the brain, suggesting that H102 peptide cannot permeate the BBB. In contrast, nasal administration of peptide solution or its liposome formulations led to significant brain uptake of H102, mainly found in the olfactory bulb among four brain sections investigated (cerebrum, cerebellum, hippocampus, and olfactory bulb) [485]. The administration of H102-loaded liposomes through intranasal route significantly improved spatial learning/memory in AD rats [485].

Zhang and colleagues explored PEG-PLGA NPs for the nasal delivery of basic fibroblast growth factor (bFGF) [486]. bFGF is a single-chain polypeptide of 155 amino acids, able to promote the survival and neurite growth of brain neurons in vitro and in vivo [487]. Administration of bFGF into the hippocampus could prevent neuronal damage and ameliorate learning deficits of AD rats [488], thus representing a potential agent for AD treatment. NPs designed by Zhang et al. were further conjugated with *Solanum tuberosum* lectin (STL), which selectively binds to *N*-acetylglucosamine on the nasal epithelial membrane [489]. The resulting NPs exhibited a uniform particle size of ca. 100 nm in diameter, and negative Z-potential values [486]. Biodistribution studies revealed that, when intravenously administered as a solution or NP formulation, bFGF levels were very low in all the examined brain sections (olfactory bulb, hippocampus, pallium, and striatum), indicating that bFGF is not capable of crossing the BBB. In contrast, bFGF was detected in the brain after intranasal NP administration, providing high bFGF concentrations in all the analysed brain sections, with statistical significance in hippocampus and pallium, as compared to bFGF solution. Histopathology assays also confirmed the in vivo safety of the developed formulations [486].

Rassu et al. proposed chitosan-coated and -uncoated SLNs as a nasal delivery system for nose-to-brain transport of BACE1 siRNA (5′-CUGUUAUCAUGGAGGGCUU-3′). Indeed, BACE1 is the major β-secretase responsible for Aβ amyloid generation in the brain [490]. To increase the transcellular pathway in neuronal cells, these NPs were further decorated with a 9R derivative of the RVG CPP (YTIWMPENPRPGTPCDIFTNSRGKRASNGGGGRRRRRRRRR) [490]. In all cases, these formulations showed a mean diameter in the 300–400 nm range. Uncoated NPs were negatively charged, while the presence of chitosan imparted a net positive charge to the particle surface [490]. The cellular transport of siRNA released from the SLNs was studied using Caco-2 cells. siRNA proved to permeate the monolayer to a greater extent when released from each of the studied formulations compared to bare siRNA, and more effectively from chitosan-coated SLNs [490]. Indeed, chitosan coating, which modified the surface charge and the mucoadhesivity of the system, improved the permeation ability of the siRNA through the epithelial cells [490].

Furthermore, curcumin-loaded nanoemulsions were proposed for intranasal delivery, and chitosan was added to improve the mucoadhesive properties of the formulations [491]. These nanosystems showed particle size in the 50–70 nm range associated with positive Z-potential values [491]. In vitro cytotoxicity studies performed on SK-N-SH cells revealed no toxicity for the designed nanosystems associated with enhanced release compared to drug solution. Ex vivo diffusion studies proved that chitosan-containing nanoemulsions showed the highest flux and permeation across the mucosa compared to uncoated nanoemulsions or bare curcumin solution [491].

Elnaggar and colleagues proposed monodisperse chitosan NPs for nose-to-brain delivery of piperine. These formulations showed a mean particle size around 200 nm, and a positive surface charge [492]. Piperine-loaded NPs delivered by intranasal route proved to be more efficient than the corresponding suspension form, providing improved efficacy at a lower dose [492]. In addition, these formulations significantly improved cognitive functions in AD animal models (Wistar rats). The use of NPs also proved to alleviate piperine-triggered nasal irritation without inducing brain toxicity [492].

### 6.2. Parkinson’s Disease

To improve dopamine brain delivery by intranasal administration, Cometa et al. investigated novel SLNs composed of the mucoadhesive polysaccharide glycol chitosan [493]. SLNs showed a mean diameter around 140 nm, positive surface charge, and high dopamine entrapment efficiency [493]. In vitro studies proved that dopamine-loaded SLNs were able to release the neurotransmitter continuously and in a sustained manner [493].

In a different study, Tang and co-workers formulated borneol and lactoferrin co-modified PEG-PLGA NPs (Lf-BNPs) loaded with dopamine [494]. In detail, borneol is a bicyclic monoterpene able to enhance brain penetration and the distribution of other drugs across the nasal mucosa and the BBB [495]. In vitro cytotoxicity studies indicated that the treatment with dopamine-functionalized Lf-BNPs did not produce a significant effect in SH-SY5Y and 16HBE cells. Furthermore, Lf modification increased the cellular uptake of NPs in both the investigated cell lines, and in turn, borneol modification promoted the cellular uptake specifically in 16HBE cells. In vivo pharmacokinetic investigations indicated that the brain concentration of dopamine was significantly higher after using targeted NPs compared to bare nanosystems [494]. Finally, intranasal administration of dopamine Lf-BNPs effectively alleviated the 6-OHDA-induced striatum lesion in PD rats [494].

In the context of polymeric NPs, WGA-modified PLGA NPs were also proposed for the nasal delivery of l-DOPA. NPs showed a mean diameter around 300 nm and a negative surface charge [496]. These physico-chemical properties were maintained after 6 months of storage, demonstrating the stability of the proposed nanosystems [496]. The designed formulations showed lower cytotoxicity compared to l-DOPA alone, reached high target tissue concentration, and were well tolerated in MPTP-induced PD model mice [496]. In addition, WGA-modified PLGA NPs significantly improved locomotor activity compared to free l-DOPA, if either orally or nasally administered [496].

Bi et al. developed Lf-modified rotigotine nanoplatforms based on PEG-PLA. The average diameter of NPs was less than 100 nm, but increased by approximately 60 nm when modified with Lf [497]. In vitro studies demonstrated that, after intranasal administration of Lf-targeted NPs, a continuous and slow release of rotigotine for 48 h occurred, also accompanied by a higher drug accumulation in the brain [497]. Conjugation with Lf improved rotigotine delivery in the olfactory bulb, striatum and cerebellum, the primary regions affected in PD [497]. In addition, compared to free rotigotine, which showed little cytotoxic effects, neither untargeted nor Lf-targeted NPs reduced the viability of 16HBE or SH-SY5Y cells [497].

In a subsequent study, the same research group investigated these nanosystems for their biodistribution, pharmacodynamics and neuroprotective effects after nose-to-brain delivery in a 6-OHDA rat model of PD [498]. After intranasal administration, NPs rapidly overcame the brain and exhibited an improved sustained-release compared with untargeted NPs. In addition, these formulations presented a higher efficacy in delivering rotigotine in the 6-OHDA PD rats than untargeted NPs, demonstrating a high capability to alleviate nigrostriatal dopaminergic neurodegeneration [498].

In a different and recent design, rotigotine was loaded onto chitosan NPs featured by an average particle size of ca. 70 nm and a positive surface charge [499]. Exposure of these formulations for 24 h did not show any cytotoxicity towards SH-SY5Y cells. Furthermore, treatment with these NPs caused a decrease in α-synuclein and an increase in tyrosine hydroxylase expression in the tested cells, suggesting that the treatment alleviated some of the direct neurotoxic effects of 6-OHDA [499]. Behavioural and biochemical testing in haloperidol-induced PD rats showed a reversal of catalepsy, akinesia, and the restoration of swimming ability [499].

Karavasili and colleagues investigated polymer-lipid microparticles loaded with ropinirole for nasal delivery. These microparticles were composed of PLGA, DPPC and trimethylchitosan (TMC), showed sizes in the 2.09–2.41 μm range and a negative surface charge [500]. Ex vivo studies demonstrated a ca. 2.4-fold enhancement of ropinirole permeation across nasal epithelium when the drug was co-formulated with TMC. In addition, all the tested formulations were found to be non-toxic in cultured human airway epithelial (Calu-3) cells [500].

More recently, ropinirole was loaded onto chitosan-coated and uncoated PLGA NPs [501]. These NPs exhibited a spherical shape and a mean average size of ca. 100 and 500 nm in diameter, for PLGA and chitosan-coated PLGA NPs, respectively. In addition, PLGA NPs showed negative Z-potential values due to the carboxyl groups of PLGA, while chitosan coating determined a net change to positive values for the amino groups of chitosan [501]. Chitosan-coated PLGA NPs showed a complete release of the drug in simulated nasal electrolyte solution over 24 h, producing a 3-fold increase of ropinirole permeation through sheep nasal mucosa, in comparison to bare PLGA NPs [501]. None of the tested formulations induced hemolysis in blood or produced ROS in Raw 264.7 cells [501].

Chitosan NPs were also used for the nose-to-brain delivery of a different dopamine agonist, i.e., pramipexole. Optimized NPs were composed of chitosan and sodium tripolyphosphate, exhibiting an average diameter around 300 nm, and a positive surface charge [502]. Their diffusion across goat nasal mucosa was very high after 24 h. Pharmacodynamic studies in rotenone-induced PD rat models revealed reduced motor deficit for pramipexole-loaded NPs as compared to free drug administration [502]. NPs also enhanced antioxidant status, increasing superoxide dismutase and catalase activities, along with increased dopamine levels in the brain [502].

Chitosan-coated PLGA NPs were also explored for the brain delivery of rasagiline [503]. These nanosystems showed a mean particle size around 120 nm in diameter, and high drug encapsulation efficiency [503]. Pharmacokinetic studies in Wistar rats proved that nasal administration of these formulations sensibly increased drug concentration in the brain and plasma compared to intravenous administration of NPs or nasal administration of the free drug solution [503].

In another study, a different lectin derivative, i.e., odorranalectin (OL), able to specifically bind L-fucose on the olfactory epithelium [504], was investigated for the targeted delivery of urocortin, exploiting PLGA-based polymeric NPs [505]. In particular, for OL conjugation, the maleimide group of PEG-PLGA was reacted with the thiol group of the lectin derivative, providing final formulations of ca. 100 nm size [505]. OL modification increased the brain delivery of NPs and enhanced the therapeutic effects of urocortin in 6-OHDA-induced hemiparkinsonian rats following intranasal administration [505].

Chitosan NPs were also recently explored for the nasal administration of naringenin, a flavonoid with significant antioxidant properties [506]. NPs showed an average diameter around 90 nm and positive Z-potential values [507]. In vitro drug release profile indicated a controlled naringenin release from the NPs in SH-SY5Y cells. The amount of drug able to permeate the nasal mucosa was dramatically higher when administered as a nanoformulation compared to the free flavonoid solution [507]. Naringenin-loaded NPs also showed enhanced neuroprotective ability and antioxidant activity against SH-SY5Y cells, without showing toxicity [507].

In a different design, resveratrol was loaded in nanoemulsions, composed of vitamin E and Sefsol (a propylene glycol mono caprylic ester) as oil phase, PS80 as a surfactant, and transcutol P as co-surfactant. Optimized formulations showed spherical globules with an average diameter of ca. 100 nm, and a negative surface charge [508]. The negligible change in Z-potential and particle size upon storage assured a long shelf life up to 3 months. Resveratrol maintained its potent antioxidant activity even when encapsulated in the nanoformulation [508]. These NPs proved to have high trans-nasal mucosal flux on the porcine nasal mucosa. Pharmacokinetic and brain-targeting studies performed on Wistar rats demonstrated a higher concentration of the drug in the brain after nasal nanoemulsion administration. Furthermore, histopathological studies on the brain sections showed decreased degenerative changes after nanoemulsion treatment [508].

In a recent investigation, the anti-inflammatory properties of geraniol (GER) with the mitochondrial rescue effects of ursodeoxycholic acid (UDCA) were combined in a newly designed prodrug, named GER-UDCA, a potential candidate against PD [509]. GER-UDCA was successfully synthesized and characterized in vitro for its ability to release the active compounds in physiological environments. Because of its poor solubility, GER-UDCA was entrapped into both SLNs and polymeric NPs, obtaining nanocarriers with an average diameter below 200 nm, and negative Z-potential values [509]. Since SLNs exhibited a higher GER-UDCA dissolution rate, this formulation was selected for subsequent in vivo experiments. Nasal administration of GER-UDCA-SLNs allowed to detect the prodrug in rat cerebrospinal fluid, but not in the bloodstream, thus suggesting a direct nose-to-brain delivery of the prodrug. In addition, nasal administration of these formulations did not damage the structural integrity of the nasal mucosa, in contrast to that observed for pure GER [509].

The nasal delivery of glial-derived neurotrophic factor (GDNF) through liposomes was investigated by two different research groups [510,511].

Bender and colleagues proposed liposomes composed of 1,2-dioleoyl-*sn*-glycero-3-phosphocholine (DOPC), Chol and stearylamine (SA) [510]. Following intranasal administration of GDNF-loaded liposomes, the concentration of the drug in the brain and olfactory bulb increased significantly within 1 h following a single dose [510]. In addition, liposomes proved to deliver GDNF 10-fold more efficiently to the brain than its pure solution, despite yielding similar neuroprotective efficacy in the 6-OHDA model. These results suggested an incomplete release of GDNF from liposomes in tissues, or the need for active targeting to specifically guide the drug [510].

Thus, in a subsequent study, Hernando et al. investigated liposomes—consisting of chitosan-coated NLCs—further decorated with the TAT peptide covalently conjugated to the liposome surface. These formulations showed a mean diameter around 200 nm and a positive surface charge [511]. In vivo studies were performed in MPTP-induced PD mouse models and revealed a significant motor recovery after intranasal NP administration [511].

NLCs were also explored for the intranasal delivery of bFGF. In particular, Zhao and colleagues proposed novel gelatin nanostructured lipid carriers (GNLs) composed of the non-ionic copolymer-poloxamer 188 and solid lipids [512]. bFGF-functionalized GNLs showed particle size around 140 nm, a strongly negative surface charge, and high drug encapsulation efficacy [512]. Intranasal administration of these formulations efficiently enriched exogenous bFGF in the olfactory bulb and striatum, without adverse impact on the integrity of nasal mucosa, also showing improved therapeutic effects on 6-OHDA induced hemiparkinsonian rats [512].

**Table 2:** Nanosystems for nose-to-brain drug delivery in the treatment of neurodegenerative diseases.

Disease	Nanocarrier Platform	Composition	Bioactive Agent	Active Targeting Ligand	Ref.
Alzheimer	Nanosuspensions	Chitosan	Donepezil, cholinesterase inhibitor		[473]
	Liposomes	DSPC/Chol/PEG (1:2:0.5 molar ratio)	Donepezil, cholinesterase inhibitor		[474]
	Liposomes	Chol and EYPC, partly enriched with α-tocopherol and/or omega-3 fatty acids	Tacrine, cholinesterase inhibitor		[475]
	Albumin NPs	β-CD, hydroxypropyl β-CD or sulphobutylether β-CD	Tacrine, cholinesterase inhibitor		[476]
	Liposomes	EPC/Chol/DSPE (1:1:0.06 molar ratio)	Rivastigmine, cholinesterase inhibitor	CPP: GLPRRRRRRRRR	[477]
	Nanoemulsions	Capmul MCM (oil phase), PS80 (surfactant), transcutol P (co-surfactant)	Rivastigmine, cholinesterase inhibitor		[478]
	Polymeric NPs	Chitosan	Rivastigmine, cholinesterase inhibitor		[479]
	Liposomes	Soy PC/Chol (30:0.2 molar ratio)	Galantamine, cholinesterase inhibitor		[480]
	Polymeric NPs	PLGA NPs	R-flurbiprofen (or tarenflurbil), anti-inflammatory		[481]
	SLNs	Glyceryl monostearate/stearic acid/soya lecithin (8:2.5:3.5 molar ratio) and PS20 (surfactant)	R-flurbiprofen (or tarenflurbil), anti-inflammatory		[481]
	Polymeric NPs	PEG-PLA NPs	VIP peptide	Wheat germ agglutinin	[483]
	Liposomes	EPC/DSPE-PEG_2000_/Chol (20:1:5 molar ratio)	H102 peptide		[485]
	Polymeric NPs	PEG-PLGA NPs	bFGF	*Solanum tuberosum* lectin	[486]
	SLNs	Chitosan-coated and uncoated SLNs composed of Witepsol E 85 solid triglycerides	BACE1 siRNA	RVG-9R	[490]
	Nanoemulsions	Mixture of Capmul MCM and Captex 500 (oil phase), Cremophor EL and PS80 (surfactants); PEG_400_ and transcutol P(co-surfactants)	Curcumin		[491]
	Polymeric NPs	Chitosan with Poloxamer 188 as stabilizer	Piperine		[492]
Parkinson	SLNs	Glycol chitosan with 0.01% of PS85	Dopamine		[493]
	Polymeric NPs	PEG-PLGA NPs	Dopamine	Lactoferrin and borneol	[494]
	Polymeric NPs	PLGA NPs	l-DOPA, dopamine precursor	Wheat germ agglutinin	[496]
	Polymeric NPs	PEG-PLGA NPs	Rotigotine, dopamine agonist	Lactoferrin	[497,498]
	Polymeric NPs	Chitosan	Rotigotine, dopamine agonist		[499]
	Microparticles	PLGA/DPPC/TMC	Ropinirole, dopamine agonist		[500]
	Polymeric NPs	Chitosan-coated and uncoated PLGA NPs	Ropinirole, dopamine agonist		[501]
	Polymeric NPs	Chitosan and sodium tripolyphosphate (6:1 molar ratio)	Pramipexole, dopamine agonist		[502]
	Polymeric NPs	Chitosan-coated PLGA NPs	Rasagiline, MAO-B inhibitor		[503]
	Polymeric NPs	PEG-PLGA NPs	Urocortine	Odorranalectin	[505]
	Polymeric NPs	Chitosan	Naringenin		[507]
	Nanoemulsions	vitamin E/Sefsol (oil phase, 1:1 molar ratio), PS80 (surfactant) and transcutol P (co-surfactant)	Resveratrol		[508]
	Polymeric NPs	PLGA NPs	GER-UDCA		[509]
	SLNs	ATO 888/Span 85 (3:1 molar ratio)	GER-UDCA		[509]
	Liposomes	DOPC/Chol/SA (50:30:5 molar ratio)	GDNF		[510]
	NLCs	Precirol ATO/Mygliol (1:1 molar ratio) as lipids, PS80 and poloxamer 188 as surfactants	GDNF	TAT peptide	[511]
	NLCs	2% gelatin solution and 20% poloxamer 188	bFGF		[512]
Huntington	Nanoemulsions	Capmul MCM (oil phase), PS80 (surfactant) and transcutol P (co-surfactant)	Tetrabenazine		[513]
	SLNs	Glycerol monostearate (lipid), PS80 and soya lecithin (surfactants), HSPC as stabilizer	Rosmarinic acid		[514]
	Liposomes	PC	Cholesterol		[515]
	Polymeric NPs	Chitosan	siRNA		[516]

### 6.3. Huntington’s Disease

In HD treatment, tetrabenazine-loaded nanoemulsions for intranasal administration were reported [513]. These formulations showed an average diameter around 100 nm, and a negative surface charge [513]. Ex vivo drug permeation studies proved that nanoformulations improved drug permeation on neuro-2a cell lines by ca. 2-fold as compared to bare tetrabenazine suspension [513]. Pharmacokinetic studies carried out in Wistar albino rats provided significantly higher drug concentration in the brain and plasma for intranasally administered tetrabenazine nanoemulsions in comparison to intravenously administered tetrabenazine solution [513]. Moreover, histopathological studies proved that nanoemulsion administration did not alter the integrity of porcine nasal mucosa [513].

Bhatt et al. investigated the use of SLNs as a drug delivery system to enhance the brain-targeting efficiency of rosmarinic acid through the intranasal administration route. The mean size of RA-loaded SLNs was found to be around 150 nm, accompanied by negative Z-potential values [514]. NP treatment significantly improved behavioural abnormalities in 3NP-treated rats in terms of body weight, beam walk, locomotor, and motor coordination and proved to significantly attenuate 3NP-induced striatal oxidative stress [514]. On the other hand, treatment with the designed formulations significantly attenuated lipid peroxidation, nitrite concentration, and restored endogenous antioxidants enzyme (catalase and GSH) activities on 3-NP-treated animals. The nasal delivery produced a higher brain concentration of the drug, as compared to the intravenous administration of NPs [514].

In a recently developed approach, Passoni and colleagues evaluated the nose-to-brain delivery of cholesterol-loaded PC-based liposomes and verified the ability of exogenous cholesterol to rescue the HD phenotypes [515]. In detail, the authors prepared liposomes loaded with deuterium-labelled cholesterol (Chol-D6) to distinguish and quantify the exogenous cholesterol from the native one [515]. They also developed a liquid chromatography–mass spectrometry (LC-MS) method to quantify the levels of Chol-D6 in brain areas and plasma after single or repeated intranasally administered doses. Chol-D6 reached measurable levels in the brain, where it persisted at least for 72 h for the slow elimination rate of cholesterol. Chol-D6 distributed and accumulated in the striatum, cortex and cerebellum, indicating the involvement of both olfactory and trigeminal pathways [515].

In a different design, the nose-to-brain delivery of chitosan NPs loaded with anti-HTT siRNA was studied in a transgenic YAC128 mouse model of HD. Chitosan/siRNA complexes formed NPs with an average size around 200 nm and a positive surface charge [516]. Intranasally administration of naked siRNA did not significantly reduce brain HTT mRNA expression. In contrast, encapsulation of siRNA in chitosan protected the drug from degradation, and the resulting NPs proved to be effective in lowering HTT mRNA expression by at least 50% [516].

### 6.4. Clinical Trials

To the best of our knowledge, the US National Institute of Health database reports only two clinical trials using nanoformulations for the treatment of AD (ClinicalTrials.gov Identifier: NCT03806478; searching for: “Alzheimer’s Disease” and “nanoparticles”) and PD (ClinicalTrials.gov Identifier: NCT03815916, searching for: “Parkinson Disease” and “nanoparticles”).

NCT03806478 is a Phase 2 study focused on the evaluation of safety, tolerability and efficacy of intranasal nanoparticles loaded with APH-1105, an alpha-secretase modulator developed by Aphios for mild to moderate cognitive impairment of AD.

NCT03815916 is a Phase 2 study to assess, via magnetic resonance spectroscopy, the effects of gold nanocrystals on the bioenergetic improvement of impaired neuronal redox state in PD patients.

## 7. Conclusions

The increasing frequency of disabling and currently incurable neurodegenerative diseases is likely to have a more and more devastating impact on individuals, families, and societies, unless effective means are found to reduce the incidence and progression of these diseases. The growth of the world’s population has been accompanied by a progressive increase in the number of elderly people. Neurodegenerative diseases, such as Alzheimer’s, Parkinson’s or Huntington’s disease, manifest themselves in adulthood with symptoms that develop insidiously and progress slowly. A long preclinical progression is described for most NDs, as pathological changes at the molecular and cellular level precede the clinical onset by several years.

Currently, there are no effective neuropreventive, neuroprotective or neurorestorative therapeutic options. However, there are good reasons to believe that these would be particularly effective in the pre-symptomatic phase of the disease, and that this phase would therefore also be ideal for prospective clinical trials.

Aiming at halting the progression of NDs, or at least alleviating their symptoms, numerous therapeutic agents have been proposed in the last decade, but these proved to be often ineffective, due to their poor accessibility to the brain.

Indeed, the BBB protects the CNS from harmful substances, restricting and controlling the permeability of various therapeutic agents and thus making their efficient brain delivery very challenging.

Advances in biotechnology are gaining a significant impact in neurological research, leading to the development of more targeted and effective therapeutic modalities. Nanotechnology represents the core of these advances, offering exciting opportunities to obtain significant achievements in the diagnosis and therapy of nervous system dysfunctions.

Nanotechnology offers a potential solution for tackling obstacles for brain delivery, providing different, novel platforms which have been proven to have great potential for the treatment of neurodegenerative disorders.

Nanoengineered delivery systems are able to avoid unfavourable immune activation and prolong the half-life of loaded cargoes, helping them to reach the brain more efficiently.

Being able to overcome drawbacks related to conventional therapeutic approaches, nanosystems have attracted growing interest for the brain delivery of drugs, and in the context of NDs, a growing number of effective nanoformulations has been successfully developed.

In addition, nanocarriers can be suitably engineered to carry multiple agents with specific functions, such as brain targeting agents, and, for this reason, several peptide- or oligonucleotide-based ligands have been employed for the specific recognition of brain cells, tissues or signalling systems.

More recently, new routes for nano-based drug delivery systems have been explored, and the nasal route as an alternative to oral and parenteral routes has gained increasing attention as a promising way to improve the access to the brain. Therefore, many efforts have been devoted to the development of nanoformulations aiming at effective nose-to-brain drug delivery.

In conclusion, nanoengineered particles represent extremely useful tools for safe, effective, target-oriented and sustained delivery across the BBB, representing a new promising hope for the treatment of neurological disorders. The scientific collaboration between the main players of research in this scientific field should help in defining future directions towards the improvement and, finally, extensive clinical application of nanomedicine-based approaches to NDs.

## Data Availability

Not applicable.
